# Environmental plasticity and colonisation history in the Atlantic salmon microbiome: A translocation experiment

**DOI:** 10.1111/mec.15369

**Published:** 2020-02-20

**Authors:** Tamsyn M. Uren Webster, Deiene Rodriguez‐Barreto, Giovanni Castaldo, Peter Gough, Sofia Consuegra, Carlos Garcia de Leaniz

**Affiliations:** ^1^ Centre for Sustainable Aquatic Research College of Science Swansea University Swansea UK; ^2^ Cynrig Fish Culture Unit Natural Resources Wales Llanfrynach UK

**Keywords:** microbiota, plasticity, priority effects, *Salmo salar*

## Abstract

Microbial communities associated with the gut and the skin are strongly influenced by environmental factors, and can rapidly adapt to change. Historical processes may also affect the microbiome. In particular, variation in microbial colonisation in early life has the potential to induce lasting effects on microbial assemblages. However, little is known about the relative extent of microbiome plasticity or the importance of historical colonisation effects following environmental change, especially for nonmammalian species. To investigate this we performed a reciprocal translocation of Atlantic salmon between artificial and semi‐natural conditions. Wild and hatchery‐reared fry were transferred to three common garden experimental environments for 6 weeks: standard hatchery conditions, hatchery conditions with an enriched diet, and simulated wild conditions. We characterized the faecal and skin microbiome of individual fish before and after the environmental translocation, using a BACI (before‐after‐control‐impact) design. We found evidence of extensive microbiome plasticity for both the gut and skin, with the greatest changes in alpha and beta diversity associated with the largest changes in environment and diet. Microbiome richness and diversity were entirely determined by environment, with no detectable effects of fish origin, and there was also a near‐complete turnover in microbiome structure. However, we also identified, for the first time in fish, evidence of historical colonisation effects reflecting early‐life experience, including ASVs characteristic of captive rearing. These results have important implications for host adaptation to local selective pressures, and highlight how conditions experienced during early life can have a long‐term influence on the microbiome and, potentially, host health.

## 1 INTRODUCTION

Microbiome structure is determined by a complex series of delicately balanced interactions with the host, the environment and amongst microbiota (Suzuki, 2017; Vellend, 2016; Walter & Ley, 2011). Unlike the host genome, the microbiome is very dynamic and readily influenced by environmental changes (Chen, Garmaeva, Zhernakova, Fu, & Wijmenga, 2018; Davenport et al., 2017). Host‐associated microbial communities are able to rapidly respond to local selective pressures due to their short generation times, rapid mutation rates, large population sizes and high levels of phenotypic plasticity and intracommunity gene flow (Walter & Ley, 2011). Given the critical and wide‐ranging influence of the microbiome on host health and fitness (Davenport et al., 2017; Koskella, Hall, & Metcalf, 2017), this extensive microbiome plasticity may also influence host tolerance of environmental challenges, or even contribute to population‐level divergence and local adaptation (Alberdi, Aizpurua, Bohmann, Zepeda‐Mendoza, & Gilbert, 2016; Louca et al., 2018; Suzuki, 2017; Walter & Ley, 2011). For example, intestinal microbiota can enhance the digestion of novel food sources and the metabolism of dietary toxins, increase drought and thermal tolerance, and increase resistance to local pathogens (Alberdi et al., 2016; Chevalier et al., 2015; Macke, Callens, De Meester, & Decaestecker, 2017).

Host‐associated microbial communities often show extensive plasticity in response to environmental change due to environmental selection, including host‐specific factors, as well as dispersal effects which influence local microbial availability (Costello, Stagaman, Dethlefsen, Bohannan, & Relman, 2012). However, historical processes can also affect the microbiome. The order and timing in which microorganisms arrive in the community can influence microbiome structure, even under otherwise identical environmental conditions (Costello et al., 2012; Maignien, DeForce, Chafee, Eren, & Simmons, 2014; Sprockett, Fukami, & Relman, 2018; Vellend, 2016). Existing colonisers may restrict and/or modify the ecological niches available to subsequent arrivals, influencing their establishment success (Fukami, 2015). For example, environmental stress or antibiotic treatment which disturbs the microbiome may allow resistant taxa to flourish in the absence of wider competition, delaying the restoration of the original complex community (Foster, Rinaman, & Cryan, 2017; Yassour et al., 2016). As observed in mammals, the microbial composition of fish is particularly dynamic during early development, as it is readily influenced by variation in the surrounding environment, host immunity and microbial seeding communities (Giatsis et al., 2016; Korpela et al., 2018; Lokesh, Kiron, Sipkema, Fernandes, & Moum, 2019; Stephens et al., 2016; Yan et al., 2016). This suggests that historical effects on microbiome structure due to environmental variation during early life may be particularly important (Gensollen, Iyer, Kasper, & Blumberg, 2016; Martínez et al., 2018; Sprockett et al., 2018). Stress experienced during early life can have a lasting detrimental effect on the microbiome and health of mammalian hosts (Foster et al., 2017), but conditioning the microbiome during early life to improve lasting host health and disease resistance could also have therapeutic benefits (Borre et al., 2014). However, overall, little is known about the extent to which historical effects may shape the microbiome, especially for nonmammalian species.

Atlantic salmon (*Salmo salar*) is one of the most commercially important fish species, and is also a keystone species for freshwater habitats (Griffiths et al., 2011). Populations of Atlantic salmon display local adaptations, and are often threatened in their natural range (Garcia de Leaniz et al., 2007). The Atlantic salmon gut and skin microbiome is known to be strongly influenced by environmental conditions, especially diet, salinity and season, as well as developmental stage (Dehler, Secombes, & Martin, 2017; Gajardo et al., 2016; Llewellyn et al., 2016; Lokesh & Kiron, 2016; Schmidt, Smith, Melvin, & Amaral‐Zettler, 2015; Zarkasi et al., 2014), and there is also substantial microbiome variation between salmon populations, especially between wild and hatchery‐reared fish, reflecting diet and environmental differences (Uren Webster, Consuegra, Hitchings, & Garcia de Leaniz, 2018). This suggests that there may be considerable microbiome plasticity in response to environmental variation. However, we also hypothesise that lasting historical effects may persist in the Atlantic salmon microbiome, reflecting early life experience and colonisation history. Understanding the scope for plasticity and historical effects is essential for the management of this species both in aquaculture and the wild. For example, microbiome plasticity and/or historical effects may influence acclimation to environmental challenges, act as a driver of local adaptation, influence survival of hatchery‐released fish, or provide a potential mechanism for improving disease resistance in aquaculture. However, little is known about the scope for plasticity following environmental change, or whether historical colonisation effects may continue to influence microbiome diversity and structure. Therefore, to investigate this, we reciprocally translocated salmon fry between hatchery and natural conditions, and employed a BACI (before‐after‐control‐impact) experimental design to test for gut and skin microbiome plasticity related with development and environment/diet, as well as potential lasting historical signatures of origin. We hypothesized that there would be a large degree of microbiome plasticity following environmental change, but that historical colonisation effects, reflecting early life experience, would also persist in the salmon microbiome.

## 2 MATERIALS AND METHODS

### 2.1 Reciprocal translocation experiment

Sixty wild Atlantic salmon fry (approximately 6 months post hatch) were captured using electrofishing from the Aber Bran, a tributary of the river Usk (Wales; lat.: 51.954, long.: –3.477) and transported a short distance (~8 km) to the Natural Resources Wales Cynrig Fish Culture Unit (Brecon, Wales). Sixty Atlantic salmon fry (6 months post hatch) were also obtained from the Cynrig Fish Culture Unit (hatchery); these fish originated from a 3:3 male:female cross between wild‐caught parents from the river Taff (Wales) that had been maintained under hatchery conditions. Hatchery fish were adipose fin clipped to differentiate them from wild fish, a procedure that does not cause adverse effects (Roberts, Taylor, Gough, Forman, & Garcia de Leaniz, 2014). All wild and hatchery fish were measured (fork length) and photographed using a Canon DS126151 400D EOS digital camera, with an 18–55 mm lens. A sample of skin‐associated mucus was collected by swabbing the left side of each fish back and forth along the entire length of the lateral line five times using Epicentre Catch‐All Sample Collection Swabs (Cambio), and gut samples were collected by gently pressing the abdomen of each fish and swabbing the expelled faeces. Fifty ml water samples were also collected from the river and hatchery. All samples were stored at –80°C prior to DNA extraction.

**Figure 1:**
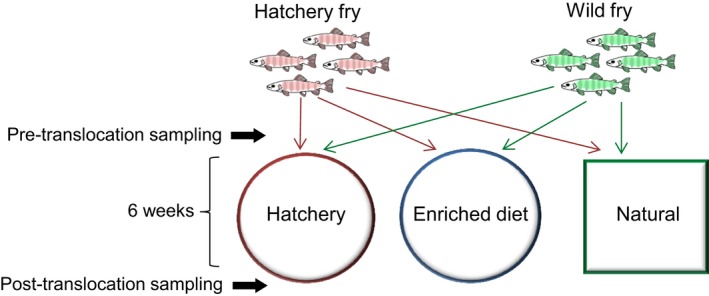
Before‐after‐control‐impact (BACI) design. Hatchery‐ and wild‐origin salmon fry were translocated to three experimental environments, employing a common garden design. Individual fish were matched at the pre‐ and post‐translocation sampling based on unique pigmentation marks [Colour figure can be viewed at http://wileyonlinelibrary.com]

Twenty wild and 20 hatchery individuals were randomly assigned to each of the three experimental environments (hatchery, enriched diet, natural) using a common garden design (Figure 1). The first experimental group was maintained in standard hatchery conditions. Fish were housed in a 500 L black plastic tank supplied with aerated, flow‐through, filtered river water (~10 L/min) and fed a standard commercial Salmonid feed (Skretting) at a rate of 3% bodyweight/day. The second group were housed in identical conditions to the first group, but were fed the standard hatchery diet enriched with daily addition of 5 g of an invertebrate mix (33% natural invertebrates harvested from the Afon Cynrig, 33% bloodworm and 33% daphnia [both JMC Aquatics]). The third experimental group consisted of near natural conditions, whereby salmon were transferred to a leat of the Afon Cynrig, another tributary of the river Usk (lat: 51.928, long: –3.358). The leat consisted of a 30 m long, 2.5 m wide diversionary watercourse from the river, isolated by upstream and downstream fry screens to prevent fish movement. It contained natural substrate, was fed by natural river water only, and received no dietary supplementation. The experimental treatments lasted for 6 weeks, after which fish were recaptured, and sampled for a second time using an identical procedure to the one described for the first sampling point (photograph, fork length, weight, skin swab, faecal swab). Fifty ml water samples were collected from the hatchery tank, enriched tank and leat. Water temperature in each of the experimental tanks and the leat ranged from 13.5 to 16.5°C during the experiment, reflecting ambient conditions.

Photographs were used to visually match individual fish at the first and second sampling points, allowing us to conduct a matched before‐and‐after microbiome analysis. Each fish was identified based on the number, shape and spacing of its parr marks, as juvenile salmonids can be identified based on a unique pattern of pigmentation (Donnelly & Whoriskey, 1993; Garcia de Leaniz, Fraser, Mikheev, & Huntingford, 1994). All matches were independently corroborated by two researchers, and eight individuals from each of the six experimental groups (wild to hatchery; hatchery to hatchery; wild to enriched; hatchery to enriched; wild to natural; hatchery to natural) were positively identified at both time‐points and used for further analyses. Specific growth rate ((Ln(length_2_) − Ln(length_1_))/time × 100; [Hopkins, 1992]) and Fulton's condition factor ([weight/length^3^] × 100; [Froese, 2006]) were calculated for each individual fish.

### 2.2 16S rRNA amplicon sequencing and bioinformatics

Microbiome analysis was performed for eight individual fish per experimental group (from the initial 20). This sample size was determined by the number of fish that were successfully recaptured from the leat and positively identified at both time points, to ensure a balanced sample design. Therefore, we analysed the skin and gut samples of 48 individual fish at each of the two time‐points (192 in total), as well as five water samples. DNA was extracted from the skin mucus and faecal samples using the MoBio PowerSoil^®^ DNA Isolation Kit (Qiagen) according to the manufacturer's instructions, with an additional incubation step of 10 min at 65°C prior to bead beating. Water samples were centrifuged at 5,000 *g* for 1 hr at 4°C and the DNA was extracted from the pellet using the same method. Briefly, 16S library preparation was performed using the primers 341F and 785R, amplifying the V3–V4 hypervariable regions of the 16S rRNA gene (Klindworth et al., 2013) selected to minimize nontarget amplification of Atlantic salmon. PCR conditions, product purification and indexing were as described previously (Uren Webster et al., 2018). All libraries were sequenced across two lanes of an Illumina MiSeq (2 × 300 bp).

Sequence data were analysed using DADA2 (Callahan et al., 2016) within Qiime2 (v2019.1, [Bolyen et al., 2018]). Briefly, all reads were first truncated to 280 bp (forward reads) and 240 bp (reverse reads), based on overall quality scores, while the first 8 bp were removed to eliminate potential adaptor contamination. Reads were then denoised, merged, subject to chimera screening and removal, and assigned into actual sequence variants (ASVs) using DADA2. Taxonomic classification of ASVs was performed within Qiime2 using the Silva reference taxonomy (v132; [Quast et al., 2013]) with a custom trained classifier (Bokulich et al., 2018), and mitochondrial, eukaryote and chloroplast sequences were removed. For selected Mycoplasmataceae ASVs, sequence alignment (ClustalW) and phylogenetic analysis (maximum likelihood method) was performed within MEGA X using default settings (Kumar, Stecher, Li, Knyaz, & Tamura, 2018). All gut, skin and water samples were subsampled to an equal depth of 16,715 reads, before calculation of alpha and beta diversity metrics (Chao1 richness [Chao, 1984], Shannon diversity [Shannon, 1948], and Bray‐Curtis dissimilarity [Bray & Curtis, 1957]).

### 2.3 Statistical analysis

All statistical analyses were performed using R (v3.4.3; [R Core Team, 2014]). To investigate differences in the specific growth rate (SGR) of individually identified fish during the course of the translocation experiment, and in final condition factor (K), linear models including the fixed factors environment (hatchery, enriched, natural), origin (hatchery, wild), and their interaction, were constructed. The most plausible models were selected based on Akaike Information Criterion (AIC) values using the step function (Table S1).

To identify initial differences in alpha diversity (Chao1 richness and Shannon diversity) at the pretranslocation sampling point, linear models were constructed including origin (hatchery/wild) and fish size (length) as fixed factors. The effects of experimental environment (hatchery/enriched/natural), origin, an environment:origin interaction and SGR on the specific change in alpha diversity for matched individuals during the course of the translocation experiment were also examined. Finally, linear models were constructed to investigate the effect of environment, origin, an environment:origin interaction and size (length), as well as the presampling alpha diversity value for matched individual fish, on final (post‐translocation) alpha diversity values (Chao1 richness and Shannon diversity). In each case reduced linear models were selected based on a lowest AIC value using bidirectional stepwise selection (Table S1). In addition, paired *t* tests were performed to further investigate the relationship between pre‐ and post‐translocation measures of alpha diversity for each individually matched fish.

Microbiome structure (beta diversity), based on Bray‐Curtis distance, was visualised using nonmetric multidimensional scaling (NMDS) ordination, using the Vegan package (Oksanen et al., 2017) then plotted using ggplot2 (Wickham, 2009). Multivariate statistical analysis of community separation (PERMANOVA) was performed using Adonis in the Vegan package. Initial (pretranslocation) differences in microbiome structure were investigated, including origin (wild/hatchery) and fish size (length) as fixed factors in the model. For the post‐translocation sampling point, the effects of environment (hatchery/enriched/natural), origin, an environment:origin interaction and fish size on final microbiome structure were tested. The specific change in gut and skin community structure (Bray‐Curtis distance) over time for individually‐matched fish was also investigated using the model (ΔBC ~ environment + origin + environment:origin + SGR). As before, the full models were reduced using stepwise simplification (Table S1).

Statistical analysis of ASV abundance was performed using DeSeq2 (Love, Huber, & Anders, 2014), using rarefied data as recommended for microbiome libraries with large deviance in total library size between samples (Weiss et al., 2017). For the gut and skin separately, the effect of origin on initial (pretranslocation) ASV abundance was examined, while the effect of environment and origin on final (post‐translocation) ASV abundance was identified using a multifactorial design including the main effects of environment, origin and their interaction. Within the DesSeq2 models, independent filtering of low coverage ASVs was applied, optimising power for identification of differentially abundant ASVs at a threshold of *α* = .05. Default settings were applied for outlier detection and moderation of ASV level dispersion estimates. ASV abundance was considered significantly different at FDR < 0.05. Heatmaps illustrating the relative abundance of the ASVs across all samples were generated using Pheatmap (Kolde, 2015) within R, based on Euclidean distance clustering.

## 3 RESULTS

### 3.1 Specific growth rate and condition

There was a significant effect of both environment (hatchery, enriched, natural) and origin (hatchery or wild) on the specific growth rate for pre/post matched individual fish, and a significant interaction between factors (*Environment* *F*_2,42_ = 55.62, *p* < .001; *Origin* *F*_1,42_ = 53.18, *p* < .001; *Environment:Origin* *F*_2,42_ = 5.02, *p = *.01; Figure S1). Specific growth rate was highest in the natural environment, and fish originating from the hatchery also showed a significantly higher growth rate than wild origin fish in both the hatchery and enriched groups. However, in the natural group there was no difference in growth rate between wild‐ and hatchery‐origin fish. There was no significant effect of environment or origin on final condition index (*Environment* *F*_2,42_ = 2.41, *p = *.10; *Origin* *F*_1,42_ = 0.02, *p* = .88; *Environment:Origin* *F*_2,42_ = 2.10, *p = *.14).

### 3.2 Microbial alpha diversity

**Figure 2:**
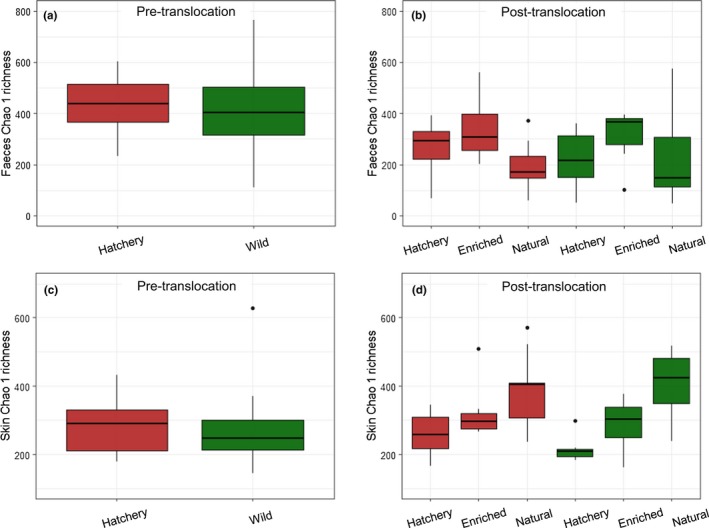
Faecal and skin Chao1 richness measure pretranslocation (a, c; *n* = 24) and post‐translocation (b, d; *n* = 8), red shading indicates hatchery origin and green shading indicates wild origin fish [Colour figure can be viewed at http://wileyonlinelibrary.com]

Water microbial richness and diversity were higher in the natural conditions than in the hatchery‐based tanks, before and after the translocation experiment (prehatchery: *Chao1* 502, *Shannon* 8.80; preriver: *Chao1* 769, *Shannon* 9.03; posthatchery‐*Chao1* 341, *Shannon* 7.49; post‐enriched‐*Chao1* 325.8, *Shannon* 7.61; post‐natural‐*Chao1* 572.2, *Shannon* 8.67). There was initially no significant difference in gut microbial richness or diversity between wild and hatchery fish (Chao1: *F*_1,46_ = 2.82, *p* = .100; Shannon: *F*_1,46_ = 1.12, *p* = .295; Figure 2a). Overall, across all matched fish, gut microbial richness and diversity significantly declined during the course of the translocation experiment (Chao1: *t*_47_ = 5.59, *p* < .001; Shannon: *t*_47_ = 5.69, *p* < .001; Figure S2). In addition, the degree of individual‐level change in gut Chao1 richness, but not Shannon diversity, was significantly affected by environment (Chao1: *Environment* *F*_2,44_ = 3.29, *p* = .046; *Origin*‐*F*_1,44_ = 1.39, *p* = .244; Shannon: *Origin* *F*_1,46_ = 2.13, *p* = .092). Fish fed an enriched diet showed a smaller reduction in gut microbial richness over time compared to those maintained in, or transferred to, a hatchery environment or a natural environment. After the translocation, final gut Chao1 richness was influenced by environment with no detectable effect of origin, size or the initial microbial richness for matched individuals (*Environment* *F*_2,44_ = 4.07, *p* = .024, *Pre‐Chao1* F_1,44_ = 2.17, *p* = .15; Figure 2b). Fish fed an enriched diet had higher gut richness than those maintained in both the hatchery and natural conditions. There was no significant effect of environment, origin or size on final gut Shannon diversity (*Origin* *F*_2,45_ = 0.67, *p* = .418, *Length* *F*_1,45_ = 3.38, *p* = .0771).

For the skin microbiome, there was no initial difference in richness or diversity between wild and hatchery fish (Chao1: *Origin* *F*_1,45_ = 0.19, *p* = .661; Shannon: *Origin* *F*_1,45_ = 0.11, *p* = .746; Figure 2c). Across all fish, there was a small reduction in skin richness and diversity over time (Chao1: *t*_46_ = −2.04, *p* = .047, Shannon: *t*_46_ = −2.29, *p* = .026), but environment had a significant effect on the degree of individual‐level change in richness and diversity during the course of the translocation experiment (Chao1: *Environment* *F*_2,40_ = 10.67, *p* < .001; *Origin* *F*_1,40_ = 2.47, *p* = .102; *SGR*_1,40_ *F* = 0.52, *p* = .473; *Environment:Origin*_2,40_ *F* = 2.39, *p* = .104; Shannon: *Environment* *F*_2,43_ = 8.52, *p* < .001; *SGR* *F*_1,43_ = 1.63, *p* = .208; Figure S3). Post‐translocation, there was also a significant effect of environment on final skin Chao1 richness and Shannon diversity (Chao1: *Environment* *F*_2,45_ = 15.11, *p* < .001; Shannon: *Environment* *F*_2,42_ = 8.62, *p* < .001; *Origin*_1,42_ = 0.55, *p* = .462; *Pre‐Shannon* *F*_1,42_ = 1.53, *p* = .22; Figure 2d). In each case, fish from the natural environment had higher richness and diversity than in both the hatchery and enriched groups, and those fed an enriched diet also showed higher richness and diversity than those in the hatchery environment. There was no detectable effect of origin, size or the pretranslocation microbial richness/diversity for matched individuals on measures of final alpha diversity in any case.

### 3.3 Microbial beta diversity

**Figure 3:**
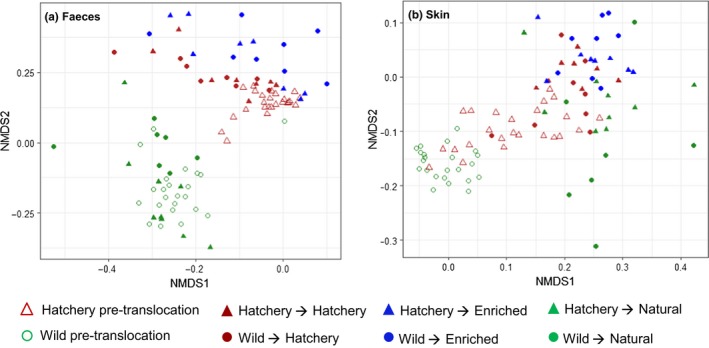
Nonmetric multidimensional scaling (NMDS) ordination of microbial community structure based on Bray‐Curtis distances, for all samples before and after the translocation experiment [Colour figure can be viewed at http://wileyonlinelibrary.com]

Before translocation, there was a significant difference in the structure of both the gut and the skin microbiome between wild and hatchery fish (Gut: *Origin* *F*_1,45_ = 26.94, *p* = .001, *Length* *F*_1,45_ = 1.05, *p* = .314; Skin: *Origin* *F*_1,44_ = 10.91, *p* = .001, *Length* *F*_1,44_ = 0.79, *p* = .581; Figure 3). Overall, during the course of the translocation experiment there was a large change in gut and skin microbiome structure across all fish, but environment and origin significantly affected the degree of structural change (Bray‐Curtis distance for individually pre/post matched fish) that occurred for both the gut and skin microbiome (Gut: *Environment* *F*_2,42_ = 3.98, *p* = .026; *Origin* *F*_1,42_ = 8.31, *p* = .006; *Environment:Origin* *F*_2,42_ = 8.30, *p* < .001; Skin: *Environment* *F*_2,43_ = 2.35, *p* = .108, *Origin* *F*_1,43_ = 28.57, *p* < .001; Figure S4). The smallest change in community structure occurred for hatchery fish maintained in the same conditions and fed the same diet, while the largest change was found in fish which experienced the greatest environmental and dietary change (i.e., wild‐hatchery and hatchery‐natural). After translocation, final skin and gut microbiome structure was strongly affected by environment as well as fish origin and fish size (Gut: *Environment* *F*_2,41_ = 6.35, *p* = .001; *Origin* *F*_1,41_ = 2.01, *p* = .039; *Length* *F*_1,41_ = 2.10, *p* = .028; *Environment:Origin* *F*_2,41_ = 1.25, *p* = .190; Skin: *Environment* F_2,41_ = 4.11, *p* = .001; *Origin* *F*_1,41_ = 3.32, *p* = .001; *Length* *F*_1,41_ = 0.91, *p* = .552; *Environment:Origin* *F*_2,41_ = 1.02, *p* = .421; Figure 3).

**Figure 4:**
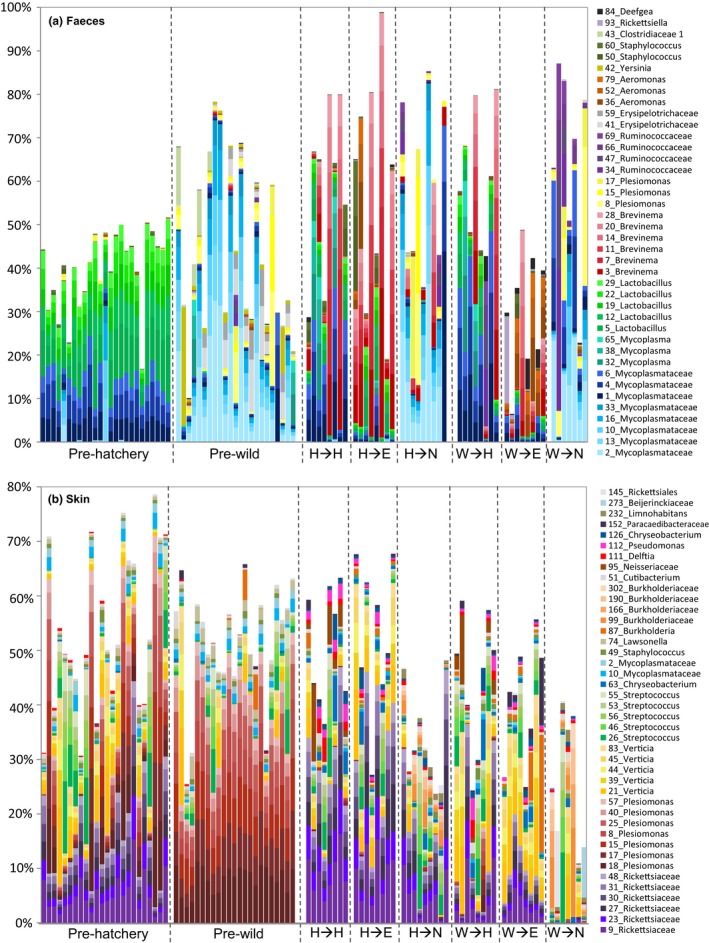
Relative abundance of the top 40 ASVs, expressed as a percentage of subsampled reads (16,715). Each bar represents an individual fish. Origin; hatchery (H) and wild (W). Experimental environment; hatchery (H), enriched (E) and natural (N) [Colour figure can be viewed at http://wileyonlinelibrary.com]

At the phylum level, the gut microbiome was dominated by Proteobacteria, Firmicutes, Terenicutes and, in some groups, Spirochaetes. In the skin microbiome, Proteobacteria were by far the most abundant bacterial phyla present, together with lower levels of Firmicutes, Actinobacteria and Bacteroidetes. The water microbiome was distinct from both the gut and the skin, with highest abundance of Proteobacteria, Bacteroidetes and Patescibacteria (Figure 4).

### 3.4 ASV abundance

**Figure 5:**
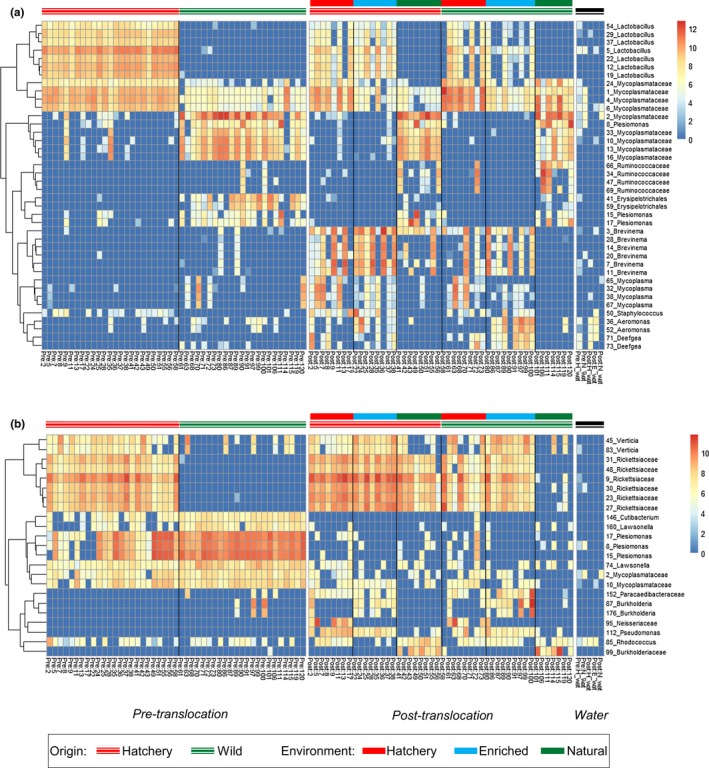
Heatmap illustrating differential abundance of ASVs in the (a) faecal and (b) skin microbiome. Data presented are log 2 transformed read counts for the top 50 ASVs which showed differential abundance between experimental environments or origin (FDR < 0.01). Hierarchical clustering was based on an Euclidean distance metric [Colour figure can be viewed at http://wileyonlinelibrary.com]

Before translocation, a total of 216 gut AVSs were significantly differentially abundant (FDR < 0.05) between wild and hatchery fish (Table S2). Notably, of the ASVs which were more abundant in hatchery fish, 47/133 (35%) were Lactobacillales. Hatchery fish were dominated by a number of *Lactobacillus* sp., as well as a cluster of ASVs from the family Mycoplasmataceae. Wild fish guts were dominated by similar, but distinct cluster of ASVs from the family Mycoplasmataceae (average 93% sequence similarity to those in hatchery fish; Figure S5), as well as number of ASVs from the family Enterobacteriaceae, including *Plesiomonas* sp. and *Yersinia* sp., and the family Erysipelotrichaceae (Figure 4a, Figure 5a). In the skin, there were initially 42 differentially abundant ASVs between wild and hatchery origin fish (Table S3). These included a number of ASVs within the family Rickettsiaceae, which were amongst the most abundant ASVs in hatchery fish (Figure 4b, Figure 5b).

After translocation, in the gut a total of 126, 243 and 283 ASVs were differentially abundant between the hatchery‐enriched, hatchery‐natural and enriched‐natural experimental groups, respectively (Table S2). In particular, the cluster of Mycoplasmataceae ASVs that were initially most abundant in wild fish were present at significantly higher levels in fish in the natural environment, while the cluster of Mycoplasmataceae ASVs initially dominant in hatchery fish were present at significantly higher levels in fish from both the hatchery and enriched environments. Additionally, fish from the hatchery environment harboured greater numbers of more than 20 *Lactobacillus* sp., and several *Mycoplasma* sp., while salmon fed the enriched diet displayed increased numbers of a number of *Aeromonas* sp. (Figure 4a, Figure 5a). In the natural environment group there were also significantly lower levels of several *Brevinema* sp. and increased abundance of a number of *Plesiomonas* sp. and ASVs within the families Ruminococcaceae and Erysipelotrichaceae, compared to fish from the hatchery and enriched groups. In addition to the effects of experimental environment, a total of 59 gut ASVs showed a significant effect of fish origin (Table S2). In particular, seven *Brevinema* ASVs were present at higher levels in hatchery‐origin fish, while notable ASVs more abundant in wild‐origin fish included several from the family Ruminococcaceae. These differences in gut community structure, reflecting both environmental treatment and origin, were also apparent in overall phyla composition. Spirochaetes, which include the genus *Brevinema,* were more abundant after translocation in fish from the hatchery and enriched environments, and in fish from a hatchery origin, but were almost entirely absent in wild fish maintained in a natural environment.

In the skin, a total of 59, 154 and 155 ASVs were differentially abundant between the hatchery‐enriched, hatchery‐natural and enriched‐natural experimental groups, respectively (Figure 4b, Figure 5b, Table S3). Notably, the six ASVs from the family Rickettsiaceae, initially more abundant in hatchery fish before the translocation, were present at significantly higher levels in both the hatchery and enriched environment groups. These Rickettsiaceae ASVs were also amongst the 32 skin ASV significantly affected by fish origin, and were more abundant in hatchery‐origin fish across all experimental environments.

## 4 DISCUSSION

We identified a near‐complete turnover of the microbiome in the Atlantic salmon skin and gut following environmental translocation, alongside developmental changes. However, in addition to this extensive microbiome plasticity we also identified some lasting effects of early life experience on microbial structure and ASV abundance. Our results, demonstrating that both environmental plasticity and historical colonisation effects determine fish microbiome assemblage, are also likely to have important implications for host health and fitness.

### 4.1 Change in the gut and skin microbiome over time

Using a powerful BACI approach, we identified a fundamental change in the gut microbiome over time. For each individually matched fish, initial gut alpha diversity had no detectable effect on final alpha diversity value after translocation and, overall, richness and diversity both significantly declined over time. Gut community structure also clearly changed over time, including a general reduction in Actinobacteria ASVs and a notable increased abundance of *Brevinema* ASVs. These differences in diversity and structure are likely to reflect developmental changes in these juvenile salmon, as well as seasonal change across the 6 week experimental period. We also observed an increase in individual microbiome variation following translocation in all experimental environments, including in the hatchery‐origin fish remaining in hatchery conditions. The teleost gut microbiome tends to become less diverse and increasingly specialised and stable as fish mature, but also more variable among individuals, reflecting a stronger influence of host‐specific factors, microbial interactions and active dispersal (Burns et al., 2016; Stephens et al., 2016; Yan et al., 2016). For the skin, there were less pronounced changes in alpha diversity over time, but considerable changes in microbiome structure including a general reduction in Alphaproteobacteria, especially Enterobacteriaceae, and increased abundance of Betaproteobacteria, as well as Actinobacteria and Flavobacteria. These results suggest that temporal dynamics in the skin and gut microbiome differ. Little is known about developmental changes in the teleost skin microbiome, but seasonality is known to be an important determinant of fish and amphibian skin microbiota (Larsen, Bullard, Womble, & Arias, 2015; Longo, Savage, Hewson, & Zamudio, 2015).

### 4.2 Microbiome plasticity in response to diet and environmental change

Before translocation, there was a clear structural distinction between the gut and skin microbiomes of hatchery‐ and wild‐origin fish, consistent with our previous results showing differences between juvenile wild and hatchery‐reared Atlantic salmon microbiomes (Uren Webster et al., 2018). The gut microbiome of wild salmon included abundant Enterobacteriaceae compared to elevated Lactobacillales in hatchery fish, while the skin of wild and hatchery fish was dominated by different groups of Proteobacteria. Following translocation, we found that microbiome richness and structure was, by far, more strongly influenced by environment than by fish origin or early‐life experience. This suggests that, at a given time, the salmon microbiome is highly dependent on the current diet and environmental conditions that a fish is exposed to, and shows considerable plasticity following environmental change. This is likely to be primarily due to environmental selection, incorporating dietary, environmental and host‐specific factors, as well as dispersal effects which restrict local microbial availability (Costello et al., 2012).

Salmon exposed to natural conditions in the leat showed significantly higher microbial richness in the skin (but not the gut) compared to fish in the hatchery and enriched environments. Water microbial richness was also higher in the natural environment, compared to the hatchery and enriched tanks. Water microbial communities are known to influence richness and diversity of the fish skin microbiome to a greater extent than the gut microbiome, where dietary diversity is thought to have a more pronounced effect (Boutin, Bernatchez, Audet, & Derôme, 2013; Giatsis et al., 2015; Uren Webster et al., 2018). While it was impossible to characterise the exact composition of the natural diet available in the leat, these results suggest that it was no more diverse than the hatchery diet. However, by enriching the standard hatchery diet with invertebrates, we were able to examine the effects of increased dietary diversity on the microbiome in isolation from other environmental variables. We found that both gut and skin microbial richness increased with dietary enrichment, but, for the skin, this was to a lesser extent than that observed in the natural environment where there was also higher water microbial richness. Furthermore, these pronounced effects of environmental translocation on microbiome richness occurred regardless of fish origin and the existing microbiome of each individual fish, demonstrating a capacity for extensive microbiome plasticity.

Regarding microbiome structure, the environment also had a very marked effect on both gut and skin microbial communities, with a clear distinction between fish from all three experimental environments. This is consistent with a strong influence of diet (e.g., Gajardo et al., 2016; Schmidt, Amaral‐Zettler, Davidson, Summerfelt, & Good, 2016) as well as water, and other environmental variables (e.g., Burns et al., 2016; Llewellyn et al., 2016; Smith, Snowberg, Caporaso, Knight, & Bolnick, 2015) on gut microbiome structure in Atlantic salmon and other fish species. Evidence from fish in the enriched group shows that dietary change specifically influences gut and, to a lesser extent, skin microbiome structure. The different bacterial communities in the water samples are also likely to affect microbiome structure, with factors influencing variable local microbial availability between the hatchery tanks and the natural environment (leat) likely to include differential environmental selection and degree of interhost dispersal. However, microbial community structure was distinct between gut, skin and water samples, indicating that salmon gut and skin microbiomes are independent and specialised communities. Overall, our results highlight that there is considerable plasticity in skin and gut microbiome structure in response to environmental change, regardless of fish origin and in spite of considerable initial differences between the wild and hatchery fish. Notably, fish subject to the largest change in diet and environmental conditions experienced the greatest change in microbiome structure, while hatchery fish maintained in hatchery conditions experienced the least.

Additionally, specific microbiome taxonomic composition was particularly distinct between fish in the natural experimental group, and those in the hatchery and enriched diet group, with a number of taxa that appeared to be distinctive of wild or hatchery conditions. In the gut we identified two clusters of highly abundant wild‐type and hatchery‐type ASVs within the family Mycoplasmataceae. A number of *Lactobacillus* sp. were also consistently enhanced in the hatchery‐based environment, while several ASVs from family Enterobacteriaceae were much more abundant under natural conditions. Notably, multiple ASVs from the genus *Brevinema* emerged only in fish translocated to the hatchery and enriched environments, suggesting it is strongly favoured by an artificial diet providing preferred metabolic substrates or growth factors, and/or other captive conditions. Similarly, in the skin microbiome, we identified a number of ASVs that appeared to be specifically enhanced in captive conditions, including from the family Rickettsiaceae, which were initially elevated in hatchery‐origin fish as well as following translocation to the hatchery and enriched environments. However, compared to the gut, there appeared to be a less pronounced effect of experimental natural and hatchery conditions on the structural composition of skin microbial communities, and fewer differentially abundant ASVs between groups. This could be because the skin microbiome is less strongly influenced by dietary change than the gut, but may also reflect the fact that the hatchery/enriched groups were supplied with filtered river water with a similar microbial profile to that of the natural environment.

### 4.3 Historical colonisation effects in the salmon microbiome

Fish origin had no discernible lasting effects on skin or gut microbiome alpha diversity, which was apparently entirely determined by environment and developmental stage. However, we did identify some persistent effects of fish origin on microbiome structure, following the 6 week translocation experiment encompassing large environmental change.

Hatchery conditions, in particular, left signatures in the gut and skin microbiome assembly regardless of environmental change, including the relative abundance of some of the most dominant community members, and in overall community structure. A number of ASVs were more abundant in hatchery‐origin fish regardless of the environment they were translocated to. In the skin microbiome, a cluster of ASVs from the family Rickettsiaceae were initially far more prevalent, and remained more prevalent, in hatchery‐origin fish translocated to each of the environmental groups. These ASVs were also strongly enhanced by the hatchery and enriched conditions, but remained very rare in fish never exposed to hatchery conditions (wild to natural group). Several gut ASVs showed similar persistent patterns in hatchery fish, including some of the most dominant community members *Mycoplasma* sp. and *Lactobacillus* sp. However, the most striking effect of origin in the gut microbiome concerned the differential emergence of *Brevinema,* a member of the phylum Spirochaetes which has also previously been identified in captive salmonids (e.g., Gupta et al., 2019; Lyons, Turnbull, Dawson, & Crumlish, 2017). *Brevinema* ASVs were only present at low levels in a small number of individuals before translocation, but they became significantly more abundant in hatchery‐origin fish across all experimental groups, even in fish translocated to natural conditions. This strongly suggests that early‐life experience of captivity promotes *Brevinema* emergence in the gut, regardless of later environmental conditions.

These effects of fish origin on community structure probably reflect differential microbial colonisation history. Priority effects describe how the order and timing of microbial arrival alters the availability of ecological niches for successive colonisers, through niche pre‐emption or modification (Costello et al., 2012; Fukami, 2015; Sprockett et al., 2018; Walter & Ley, 2011). Early colonisation of the fish microbiome depends on dispersal limitation and random sampling, that determine local microbial availability, and environmental selection (Costello et al., 2012), and was clearly different for wild and hatchery fish based on the initial observed differences in their microbiomes and between water samples. Large populations of dominant taxa, established during early development, are then likely to be able to outcompete later arrivals, and may also have an increased potential to adapt to environmental change, due to a higher probability of mutation and gene flow (Howe et al., 2015; Sprockett et al., 2018; Walter & Ley, 2011). This may account for the continued presence of dominant ASVs, including Rickettsiaceae and *Mycoplasma* sp. in hatchery‐origin fish following translocation to the natural environment. At the same time, the preferential emergence of *Brevinema* ASVs in hatchery‐origin fish could be explained by niche modification, whereby previous colonisers in the hatchery gut provide favourable nutrients or conditions for *Brevinema* growth, and/or conditions in the wild fish gut inhibit their successful colonisation (Fukami, 2015).

In addition to priority effects due to microbial colonisation history, it is likely that host‐specific differences between the wild and hatchery salmon populations may also contribute to the observed lasting signatures of origin on microbiome structure. These could include differences in the genetic background of the two fish populations. While host genotype is known to influence overall microbiome diversity and structure to a lesser extent than environmental factors (Burns et al., 2017; Stagaman, Burns, Guillemin, & Bohannan, 2017; Uren Webster et al., 2018), mammalian studies have demonstrated heritability of certain microbial taxa (Goodrich et al., 2016) and this may account for some of the differences in ASVs observed between wild and hatchery populations after translocation. However, for the main hatchery signatory ASVs identified, including Rickettsiaceae in the skin and *Brevinema* in the gut*,* their marked decline in hatchery fish transferred to the natural environment suggests their abundance was primarily environmentally, not genetically, determined. Additionally, the two fish populations are likely to have experienced very different environmental conditions and pathogens in the wild and captivity during early life, shaping the development of their adaptive immune system and, thus, the nature of selective processes influencing microbiota assembly (Foster et al., 2017; Gensollen et al., 2016). Further research is required to establish the relative contributions of host genetic and environmentally‐driven epigenetic influences on lasting signatures of origin in the fish microbiome, relative to historical microbial colonisation effects.

### 4.4 Perspective

Overall, we show that the scope for environmental plasticity in the fish gut and skin microbiome is extensive, with a near complete turnover in microbial richness and structure evident following environmental translocation. At the same time, we show, for the first time in fish, clear evidence that conditions experienced in early life can have a lasting influence on microbiome structure, for at least 6 weeks after translocation to a new environment, probably primarily due to the influence of microbial colonisation history through priority effects. These findings could have a range of implications for evolutionary ecology, conservation and management. For example, extensive metagenomic plasticity, reflecting environmental variation, could potentially influence host capacity to adapt to environmental challenges, such as climate change, emergent pathogens and pollution (Alberdi et al., 2016; Louca et al., 2018; Walter & Ley, 2011). Historical colonisation effects, potentially associated with a given host phenotype in a certain environment, could represent a mechanism contributing to local adaptation, or even phenotypic mismatch in hatchery‐released fish used to supplement natural populations (Stringwell et al., 2014). Potentially, differences in the gut microbiome of hatchery‐reared salmon, influencing digestion, nutrient uptake or metabolism, may influence the growth rate of these fish compared to their wild counterparts (Butt & Volkoff, 2019). In addition, our results demonstrate how common hatchery husbandry processes which alter microbial colonisation and succession, such as antimicrobial treatments, could have a lasting impact on the fish microbiome and, potentially, also on host health. However, they also highlight the possibility of conditioning the microbiome, for example through diet, to improve disease resistance in farmed fish or reduce phenotypic mismatch in hatchery‐released fish. Future research is required to determine whether the historical effects characterised here extend for longer than 6 weeks, and also to establish causal links between the microbiome and host phenotype. This is essential to establish the relative importance of microbiome plasticity and historical colonisation effects on host fitness.

## AUTHOR CONTRIBUTION

C.G.L., T.U.W., P.G., and S.C. designed the study; T.U.W., D.R.B., and G.C. performed the experiment; T.U.W., and D.R.B. analysed the data; T.U.W., C.G.L., and S.C. wrote the manuscript. All authors contributed to the final version of the manuscript.

## Data Availability

All Illumina sequence reads are available from the European Nucleotide Archive under the accession number PRJEB30953. Full metadata is provided in the online Supporting Information.
