# Supplementary material for: Environmental plasticity and colonisation history in the Atlantic salmon microbiome: A translocation experiment
Source: Mol Ecol. 2020 Feb 20;29(5):886–98. doi: 10.1111/mec.15369 (PMC7078932; doi:10.1111/mec.15369)
Supplement: Supplementary file 1 [file MEC-29-886-s001.pdf]

SUPPORTING INFORMATION

Environmental plasticity and colonisation history in the Atlantic salmon microbiome: a translocation experiment

Tamsyn M. Uren Webster[1], Deiene Rodriguez-Barreto[1], Giovanni Castaldo[1], Peter Gough[2], Sofia Consuegra[1], Carlos Garcia de Leaniz[1]

1. Centre for Sustainable Aquatic Research, College of Science, Swansea University, Swansea, SA2 8PP, UK
2. Natural Resources Wales, Cynrig Fish Culture Unit, Llanfrynach, Powys LD3 7AQ, UK

This supporting information contains:

P2-3: Table S1. Selection of linear models based on AIC values.

P4-13: Table S2. Differentially abundant gut ASVs.

P14-19: Table S3. Differentially abundant skin ASVs.

P20: Figure S1. Specific growth rates for individual fish during the experiment.

P21: Figure S2. Change in faecal Chao1 richness over time.

P22: Figure S3. Change in skin Chao1 richness over time.

P23: Figure S4. Change in microbiome structure.

P24: Figure S5. Phylogenetic analysis of selected Mycoplasmataceae ASVs.

Table S1. Model selection table. Selection of linear models, based on AIC (Akaike Information Criterion) values, using the *Step* function.

	Model	AIC	ΔAIC	Notes
	SGR ~ environment + origin + environment:origin	-208.50	0.00	Full model
	k ~ environment + origin + environment:origin	-216.55	0.00	Full model
	k ~ environment + origin	-215.98	0.57	Preferred model
Pre-translocation alpha diversity	F_chao_pre ~origin + pre.length + origin:pre.length	506.47		Full model
	F_chao_pre ~ origin	503.62	0.00	Preferred model
	F_chao_pre ~ origin + pre.length	504.88	1.26	
	F_shannon_pre ~ origin + pre.length + origin:pre.length	-0.71		Full model
	F_shannon_pre ~ origin	-4.49	0.00	Preferred model
	F_shannon_pre ~ origin + pre.length	-2.50	1.99	
	S_chao_pre ~ origin + pre.length + origin:pre.length	419.69	1.74	Full model
	S_chao_pre ~ origin	417.95	0.00	Preferred model
	S_chao_pre ~ origin + pre.length	419.26	1.31	
	S_shannon_pre ~ origin + pre.length + origin:pre.length	-35.72	1.62	Full model
	S_shannon_pre ~ origin	-37.34	0.00	Preferred model
	S_shannon_pre ~ origin + pre.length	-35.80	1.54	
Post-translocation alpha diversity	F_chao_post ~ environment + origin + post_length + F_chao_pre + environment:origin	469.90		Full model
	F_chao_post ~ environment + post_length + F_chao_pre	466.36	1.84	
	F_chao_post ~ environment + origin + F_chao_pre	466.13	1.61	
	F_chao_post ~ environment + F_chao_pre	464.52	0.00	Preferred model
	F_chao_post ~ environment	464.83	0.31	
	F_shannon_post ~ environment + origin + post_length + F_shannon_pre + environment:origin	35.28		Full model
	F_shannon_post ~ post_length	25.54	0.00	
	F_shannon_post ~ origin + post_length	27.22	1.68	Preferred model
	S_chao_post ~ environment + origin + post_length + S_chao_pre + environment:origin	419.98		Full model
	S_chao_post ~ environment	413.22	0.00	Preferred model
	S_chao_post ~ environment + S_chao_pre	413.84	0.62	
	S_chao_post ~ environment + origin + S_chao_pre	415.21	1.99	
	S_shannon_post ~ environment + origin + post_length + S_shannon_pre + environment:origin	-47.54		Full model
	S_shannon_post ~ environment + S_shannon_pre	-52.74	0.00	
	S_shannon_post ~ environment	-52.96	0.22	
	S_shannon_post ~ environment + post_length + S_shannon_pre	-51.22	1.52	
	S_shannon_post ~ environment + origin + S_shannon_pre	-51.69	1.05	Preferred model

Change in α diversity	DF_Chao ~ environment + origin + SGR + environment:origin	536.15		Full model
	DF_Chao ~ environment	531.06	0.00	
	DF_Chao ~ environment + origin	531.56	0.50	Preferred model
	DF_Chao ~ environment + SGR	532.53	1.47	
	DF_Shannon ~ environment + origin + SGR + environment:origin	58.25		Full model
	DF_Shannon ~ origin + SGR	51.83	1.81	
	DF_Shannon ~ origin	50.02	0.00	Preferred model
	DS_Chao ~ environment + origin + SGR + environment:origin	444.60	0.00	Full model
	DS_Chao ~ environment + origin + SGR	445.92	1.32	
	DS_Chao ~ environment + origin + environment:origin	445.18	0.58	
	DS_Shannon ~ environment + origin + SGR + environment:orign	-0.76		Full model
	DS_Shannon ~ environment	-5.78	0.00	
	DS_Shannon ~ environment + origin	-4.35	1.43	
	DS_Shannon ~ environment + SGR	-5.53	0.25	Preferred model
Change in β diversity	F_BC_distance ~ environment + origin + SGR + environment:origin	-219.00	-0.99	Full model
	F_BC_distance ~ environment + origin + environment:origin	-219.99	0.00	Preferred model
	S_BC_distance ~ environment + origin + SGR + environment:origin	-211.64		Full model
	S_BC_distance ~ environment + origin	-215.89	0.00	Preferred model
	S_BC_distance ~ origin	-214.35	-1.54	
	S_BC_distance ~ environment + origin + SGR	-213.95	-1.94	

Table S2. Differentially abundant gut ASVs.

ASV no.	Taxonomy	Pre-translocation Wild:Hatchery		Treatment Hatchery:Enriched		Treatment Hatchery:Natural		Treatment Enriched:Natural		Origin Hatchery:Wild	
		Log2 FC	FDR	Log2 FC	FDR	Log2 FC	FDR	Log2 FC	FDR	Log2 FC	FDR
3	G__Brevinema	NA	NA	0.00	1.00E+00	5.02	6.43E-10	5.02	6.68E-10	3.76	3.88E-07
7	G__Brevinema	NA	NA	0.36	8.90E-01	6.61	3.48E-11	6.25	4.63E-10	2.84	6.96E-03
11	G__Brevinema	NA	NA	0.20	1.00E+00	4.86	4.42E-06	4.66	9.53E-06	3.36	3.32E-03
14	G__Brevinema	NA	NA	-0.19	1.00E+00	4.69	1.78E-05	4.89	6.46E-06	3.11	3.90E-03
20	G__Brevinema	NA	NA	-0.41	8.80E-01	4.40	5.31E-05	4.81	7.71E-06	3.03	7.39E-03
28	G__Brevinema	NA	NA	-0.27	9.96E-01	4.44	7.91E-05	4.71	2.42E-05	2.72	1.83E-02
433	G__Brevinema	NA	NA	-2.15	2.17E-02	0.17	9.98E-01	2.32	7.88E-03	2.88	3.76E-03
65	G__Mycoplasma	NA	NA	5.83	1.13E-11	7.17	5.01E-17	1.34	1.45E-01	2.00	3.18E-02
60	G__Staphylococcus	-4.82	2.76E-13	-1.07	2.67E-01	6.19	6.75E-13	7.26	3.06E-17	1.76	4.72E-02
105	G__Staphylococcus	-2.57	4.06E-03	-1.04	2.93E-01	5.37	1.01E-09	6.41	1.58E-13	1.93	2.73E-02
23	F__Rickettsiaceae	-5.55	1.54E-19	-0.53	6.21E-01	1.89	1.75E-02	2.42	1.93E-03	2.44	3.43E-03
408	G__Lactobacillus	-4.38	5.55E-13	0.19	9.60E-01	1.69	2.19E-02	1.51	3.78E-02	2.10	3.90E-03
30	F__Rickettsiaceae	-5.14	5.98E-15	-0.56	5.75E-01	1.67	2.76E-02	2.23	2.65E-03	1.86	1.18E-02
384	G__Vagococcus	-4.58	6.97E-15	-0.83	3.04E-01	1.63	3.20E-02	2.46	5.75E-04	1.57	3.09E-02
121	G__Staphylococcus	NA	NA	-0.74	5.84E-01	4.19	1.64E-05	4.94	2.77E-07	2.19	2.48E-02
154	G__Staphylococcus	NA	NA	-1.34	1.79E-01	4.04	1.52E-05	5.38	3.90E-09	2.08	2.12E-02
217	G__Acinetobacter	NA	NA	1.12	1.27E-01	2.38	7.12E-04	1.27	8.27E-02	1.40	4.87E-02
463	G__Pseudomonas	NA	NA	1.07	1.41E-01	2.23	1.37E-03	1.15	1.10E-01	1.47	3.92E-02
16	F__Mycoplasmataceae	3.30	8.27E-03	0.00	1.00E+00	-8.15	5.25E-35	-8.15	5.12E-35	1.51	4.31E-02
89	F__Clostridiaceae 1	4.84	1.11E-09	0.63	5.75E-01	-0.42	6.78E-01	-1.05	1.64E-01	2.08	1.08E-02
10	F__Mycoplasmataceae	2.99	1.15E-01	0.57	6.33E-01	-6.73	6.86E-21	-7.30	7.36E-23	1.91	1.61E-02
48	F__Rickettsiaceae	-3.96	1.63E-10	-1.22	7.92E-02	0.84	2.78E-01	2.06	2.18E-03	1.46	3.20E-02
27	F__Rickettsiaceae	-4.83	1.66E-13	-0.96	2.44E-01	0.68	4.06E-01	1.63	1.99E-02	1.82	1.18E-02
31	F__Rickettsiaceae	-5.19	1.53E-15	-1.92	1.37E-02	0.63	5.01E-01	2.55	6.13E-04	2.15	4.43E-03
225	G__Roseomonas	NA	NA	0.54	6.50E-01	-3.77	1.59E-08	-4.31	5.47E-10	1.46	4.90E-02
240	O__Betaproteobacteriales	NA	NA	-1.04	1.84E-01	1.00	2.08E-01	2.04	4.56E-03	2.00	7.52E-03
544	O__Bacillales	NA		-2.80	1.81E-04	0.02	1.00E+00	2.82	8.25E-05	1.87	1.52E-02
66	F__Ruminococcaceae	NA	NA	3.56	6.07E-04	-4.19	3.45E-06	-7.75	1.07E-16	-3.32	5.67E-04
78	F__Ruminococcaceae	NA	NA	2.53	2.91E-02	-4.04	6.77E-05	-6.57	1.14E-10	-3.42	9.19E-04
69	F__Ruminococcaceae	NA	NA	3.52	1.28E-03	-4.04	2.87E-05	-7.56	2.23E-14	-3.16	9.19E-04
34	F__Ruminococcaceae	NA	NA	5.11	4.36E-06	-3.69	2.91E-04	-8.79	4.12E-17	-2.90	1.06E-02
47	F__Ruminococcaceae	NA	NA	4.47	8.63E-05	-3.50	7.12E-04	-7.97	4.49E-14	-3.00	6.24E-03
122	F__Ruminococcaceae	NA	NA	3.29	2.52E-03	-3.24	7.96E-04	-6.52	4.81E-11	-2.70	1.09E-02
407	G__Plesiomonas	NA	NA	0.00	1.00E+00	-3.29	1.32E-05	-3.29	1.10E-05	-2.23	4.43E-03
682	G__Rickettsia	NA	NA	0.00	1.00E+00	-2.92	6.96E-05	-2.92	6.08E-05	-1.98	1.13E-02
109	G__Enterococcus	NA	NA	0.00	1.00E+00	-2.25	1.14E-03	-2.25	1.03E-03	-1.62	2.43E-02
396	G__Romboutsia	2.60	1.04E-02	0.00	1.00E+00	-2.14	2.06E-03	-2.14	1.93E-03	-1.45	4.87E-02
168	F__Enterobacteriaceae	NA	NA	2.94	6.79E-04	5.32	6.24E-10	2.38	9.07E-03	-1.79	4.79E-02
177	G__Deefgea	NA	NA	-0.69	5.65E-01	4.61	2.32E-07	5.29	2.11E-09	-1.96	2.73E-02

137	G__Aeromonas	NA	NA	-0.36	8.44E-01	4.34	1.82E-06	4.70	1.89E-07	-2.81	9.19E-04
343	G__Deefgea	NA	NA	0.43	7.64E-01	4.30	6.67E-07	3.88	7.88E-06	-1.80	4.13E-02
244	F__Desulfovibrionaceae	NA	NA	1.04	2.29E-01	4.21	1.06E-07	3.18	6.97E-05	-2.45	3.32E-03
209	G__Deefgea	NA	NA	-0.66	5.75E-01	4.19	1.77E-06	4.85	2.34E-08	-2.06	1.66E-02
226	G__Aeromonas	NA	NA	-0.85	4.26E-01	4.07	4.57E-06	4.92	1.78E-08	-2.00	1.87E-02
77	G__Aeromonas	NA	NA	-0.63	6.47E-01	3.63	7.91E-05	4.26	2.60E-06	-2.34	1.46E-02
410	G__Deefgea	NA	NA	0.08	1.00E+00	3.63	1.94E-05	3.55	2.84E-05	-1.69	4.72E-02
84	G__Deefgea	NA	NA	-0.73	6.09E-01	2.95	2.85E-03	3.69	1.63E-04	-2.35	1.87E-02
804	G__Aeromonas	NA	NA	0.35	7.91E-01	2.68	5.88E-04	2.33	3.01E-03	-2.02	1.13E-02
583	G__Aeromonas	NA	NA	-1.65	1.26E-02	1.56	3.14E-02	3.21	2.99E-06	-1.44	4.13E-02
198	G__Paracoccus	NA	NA	-0.92	2.41E-01	1.53	3.98E-02	2.45	5.09E-04	-2.25	3.32E-03
231	G__Clostridium sensu stricto 9	NA	NA	-3.77	1.29E-06	-0.04	1.00E+00	3.73	6.48E-07	-2.51	2.06E-03
460	G__Clostridium sensu stricto 9	NA	NA	-3.09	4.36E-05	-0.03	1.00E+00	3.06	2.11E-05	-2.06	9.06E-03
194	G__Crenobacter	NA	NA	-5.09	2.60E-12	-0.03	1.00E+00	5.06	1.04E-12	-1.85	1.66E-02
625	G__Hypnocyclicus	NA	NA	-2.70	2.88E-04	-0.02	1.00E+00	2.68	1.65E-04	-1.80	1.61E-02
623	C__Gracilibacteria	NA	NA	-2.51	4.06E-04	-0.02	1.00E+00	2.49	2.29E-04	-1.68	1.87E-02
792	G__Fusibacter	NA	NA	-2.41	1.03E-03	-0.02	1.00E+00	2.39	6.41E-04	-1.61	3.18E-02
443	F__Chromobacteriaceae	NA	NA	-3.98	4.32E-10	-0.02	1.00E+00	3.96	1.97E-10	-1.56	1.87E-02
430	G__Pseudorhodobacter	NA	NA	-1.78	2.86E-02	0.37	7.49E-01	2.15	4.88E-03	-1.74	2.61E-02
281	G__Aeromonas	NA	NA	-1.77	4.43E-02	1.09	2.32E-01	2.86	5.87E-04	-1.74	4.34E-02
13	F__Mycoplasmataceae	3.28	1.26E-03	-0.11	1.00E+00	-8.55	1.09E-37	-8.44	1.85E-37	1.20	1.62E-01
17	G__Plesiomonas	3.43	2.85E-05	0.71	6.01E-01	-8.31	1.16E-25	-9.01	8.12E-28	0.72	4.73E-01
2	F__Mycoplasmataceae	3.33	4.25E-02	1.22	1.50E-01	-8.23	1.76E-35	-9.45	1.82E-43	1.10	1.74E-01
8	G__Plesiomonas	4.79	4.47E-11	0.93	3.43E-01	-8.10	8.11E-33	-9.03	4.16E-37	-0.16	9.63E-01
33	F__Mycoplasmataceae	3.41	4.06E-04	-0.66	5.36E-01	-7.58	2.25E-29	-6.92	6.44E-27	0.73	3.75E-01
15	G__Plesiomonas	3.61	3.76E-07	0.00	1.00E+00	-5.46	5.74E-17	-5.46	7.98E-17	0.00	NA
25	G__Plesiomonas	2.73	6.22E-04	-0.29	8.75E-01	-3.03	7.66E-06	-2.74	2.84E-05	-0.67	4.02E-01
18	G__Plesiomonas	2.80	4.83E-03	0.72	5.12E-01	-2.18	1.93E-03	-2.90	7.30E-05	0.10	9.86E-01
59	F__Erysipelotrichaceae	8.20	7.39E-30	0.00	1.00E+00	-2.72	3.03E-05	-2.72	2.61E-05	-0.02	NA
41	F__Erysipelotrichaceae	8.53	4.61E-36	0.79	4.53E-01	-2.70	9.70E-05	-3.48	1.35E-06	-0.03	NA
91	F__Erysipelotrichaceae	7.61	4.19E-26	-0.23	9.42E-01	-2.65	1.07E-04	-2.42	2.85E-04	0.02	NA
223	O__Rhizobiales	5.14	9.46E-25	0.35	7.77E-01	-4.62	1.50E-17	-4.98	2.39E-18	-0.50	4.66E-01
236	G__Luteolibacter	3.19	5.08E-07	0.00	1.00E+00	-4.50	3.81E-12	-4.50	4.24E-12	-0.27	8.10E-01
229	F__Rhodobacteraceae	3.03	2.20E-06	-0.90	3.43E-01	-4.41	6.65E-11	-3.52	3.40E-08	-0.64	4.39E-01
197	F__Rhodobacteraceae	3.81	2.53E-11	0.00	1.00E+00	-3.69	1.45E-07	-3.69	1.27E-07	-0.33	7.86E-01
450	G__Luteolibacter	3.19	4.10E-06	0.00	1.00E+00	-3.66	2.16E-07	-3.67	1.95E-07	-0.38	7.49E-01
402	G__Aeromonas	4.49	8.36E-10	0.00	1.00E+00	-3.52	8.54E-07	-3.52	7.32E-07	-0.58	5.60E-01
199	G__Arenimonas	2.66	3.42E-04	0.00	1.00E+00	-3.41	2.60E-07	-3.41	2.40E-07	-0.24	8.90E-01
216	G__Bacillus	2.96	3.01E-05	0.51	7.00E-01	-3.25	1.08E-05	-3.76	7.12E-07	0.24	9.13E-01
251	O__Rhizobiales	3.27	1.05E-09	-1.30	1.08E-01	-3.16	3.45E-06	-1.86	4.50E-03	-0.61	4.41E-01
388	G__SH-PL14	3.12	4.78E-09	-0.35	7.79E-01	-3.14	1.42E-07	-2.79	9.87E-07	-0.37	6.94E-01
471	G__Arenimonas	3.05	2.20E-05	0.00	1.00E+00	-3.13	1.51E-06	-3.13	1.29E-06	-0.27	8.14E-01
156	G__Carnobacterium	3.39	1.28E-05	-1.16	2.28E-01	-3.02	9.40E-05	-1.86	9.73E-03	0.68	4.46E-01
338	G__Tabrizicola	2.61	1.58E-04	-1.61	6.67E-02	-2.98	9.19E-05	-1.36	5.31E-02	-1.42	1.23E-01

348	G__Tabrizicola	3.70	2.42E-11	-0.81	4.31E-01	-2.93	5.37E-05	-2.12	1.96E-03	-0.64	4.43E-01
545	F__Microbacteriaceae	2.67	4.09E-04	-0.92	3.43E-01	-2.78	1.14E-04	-1.86	6.18E-03	-1.01	2.11E-01
188	G__Clostridium sensu stricto 1	4.62	2.39E-13	0.00	1.00E+00	-2.77	6.02E-05	-2.77	5.14E-05	-0.34	7.49E-01
319	G__Bacillus	3.21	3.08E-08	0.00	1.00E+00	-2.59	9.36E-05	-2.59	8.20E-05	-0.54	5.46E-01
317	F__Verrucomicrobiaceae	3.76	1.50E-09	0.00	1.00E+00	-2.50	1.14E-04	-2.50	1.03E-04	-0.35	NA
505	G__Bacillus	3.26	1.53E-07	0.00	1.00E+00	-2.46	1.33E-04	-2.46	1.21E-04	-0.36	NA
389	F__Rhodobacteraceae	4.76	7.62E-18	0.00	1.00E+00	-2.33	1.85E-04	-2.33	1.66E-04	-0.68	NA
440	G__Gaiella	2.67	4.38E-04	0.00	1.00E+00	-2.25	5.46E-04	-2.25	4.78E-04	-0.77	NA
456	G__Tabrizicola	3.17	6.12E-06	0.00	1.00E+00	-2.24	5.55E-04	-2.24	4.86E-04	-0.71	NA
434	G__Polymorphobacter	3.01	7.93E-06	0.00	1.00E+00	-2.22	4.33E-04	-2.22	3.85E-04	-0.34	NA
395	O__Rhizobiales	4.81	9.07E-15	0.00	1.00E+00	-2.18	6.60E-04	-2.18	5.75E-04	-0.46	NA
617	F__Rhizobiales Incertae Sedis	2.47	6.18E-04	-0.50	6.36E-01	-2.09	1.39E-03	-1.59	1.01E-02	-0.89	NA
449	F__Rhodobacteraceae	3.56	1.56E-11	0.00	1.00E+00	-2.05	9.35E-04	-2.05	8.43E-04	0.07	NA
514	G__Arenimonas	2.68	4.90E-05	0.00	1.00E+00	-2.00	8.79E-04	-2.00	7.77E-04	-0.50	NA
533	F__Gemmataceae	2.68	2.76E-05	0.23	9.09E-01	-1.96	8.94E-04	-2.20	2.73E-04	-0.02	NA
522	O__Planctomycetales	3.75	5.51E-11	0.00	1.00E+00	-1.95	2.03E-03	-1.96	1.89E-03	0.30	NA
181	G__Carnobacterium	2.80	8.06E-02	-0.94	2.99E-01	-1.77	1.32E-02	-0.84	2.06E-01	0.05	NA
561	G__Chthoniobacter	3.07	9.35E-08	0.00	NA	-1.52	1.13E-02	-1.52	1.02E-02	0.01	NA
435	F__Rhodocyclaceae	3.56	4.47E-07	0.00	1.00E+00	-1.44	2.83E-02	-1.44	2.47E-02	0.94	NA
429	G__Fodinicola	4.71	1.66E-13	0.00	NA	-1.40	2.37E-02	-1.40	2.06E-02	-0.95	NA
40	G__Plesiomonas	NA	NA	0.00	NA	-1.56	1.13E-02	-1.57	1.02E-02	-0.11	NA
57	G__Plesiomonas	NA	NA	0.00	NA	-1.46	1.70E-02	-1.46	1.52E-02	0.01	NA
125	F__Mycoplasmataceae	NA	NA	0.00	1.00E+00	-7.31	1.36E-27	-7.31	1.56E-27	0.00	NA
144	G__Roseomonas	NA	NA	-0.56	6.12E-01	-6.36	1.98E-22	-5.79	1.43E-20	0.33	7.49E-01
337	F__Mycoplasmataceae	NA	NA	0.00	1.00E+00	-5.52	8.23E-16	-5.53	9.66E-16	-0.01	NA
375	F__Beijerinckiaceae	NA	NA	0.00	1.00E+00	-4.69	6.64E-15	-4.69	7.32E-15	0.48	6.41E-01
298	F__Mycoplasmataceae	NA	NA	0.00	1.00E+00	-5.75	1.76E-14	-5.75	2.08E-14	0.13	9.75E-01
202	G__Plesiomonas	NA	NA	0.92	4.10E-01	-4.89	4.19E-11	-5.81	6.50E-14	-0.89	3.63E-01
608	F__Mycoplasmataceae	NA	NA	0.00	1.00E+00	-4.04	1.33E-07	-4.04	1.15E-07	-1.08	2.37E-01
93	G__Rickettsiella	NA	NA	-4.82	2.33E-12	-1.87	1.04E-02	2.95	7.70E-07	-0.58	4.58E-01
697	F__Gemmataceae	NA	NA	0.00	1.00E+00	-3.04	1.55E-06	-3.04	1.33E-06	0.28	8.04E-01
351	F__Rhodobacteraceae	NA	NA	0.00	1.00E+00	-3.25	1.82E-06	-3.25	1.58E-06	-0.02	NA
722	F__Beijerinckiaceae	NA	NA	0.00	1.00E+00	-2.80	3.31E-05	-2.80	2.84E-05	0.15	9.61E-01
520	F__Rhizobiales Incertae Sedis	NA	NA	0.00	1.00E+00	-2.46	5.06E-05	-2.46	4.22E-05	-0.58	NA
641	O__Betaproteobacteriales	NA	NA	0.00	1.00E+00	-2.49	1.05E-04	-2.49	9.45E-05	0.24	NA
825	O__Rhizobiales	NA	NA	0.00	1.00E+00	-2.29	3.01E-04	-2.29	2.71E-04	-0.50	NA
232	G__Limnohabitans	NA	NA	0.00	1.00E+00	-2.15	8.53E-04	-2.16	7.58E-04	0.66	NA
99	F__Burkholderiaceae	NA	NA	0.00	1.00E+00	-2.02	2.06E-03	-2.02	1.93E-03	1.32	7.08E-02
837	F__Halieaceae	NA	NA	0.00	1.00E+00	-1.80	6.59E-03	-1.81	6.14E-03	-1.22	NA
670	G__Tabrizicola	NA	NA	0.00	1.00E+00	-1.79	7.19E-03	-1.79	6.62E-03	-1.21	NA
809	G__Tabrizicola	NA	NA	0.00	1.00E+00	-1.63	1.21E-02	-1.63	1.09E-02	-1.10	NA
163	G__Enterococcus	NA	NA	0.00	1.00E+00	-1.56	1.69E-02	-1.56	1.51E-02	-1.16	NA
5	G__Lactobacillus	-9.65	1.13E-170	1.29	5.50E-02	8.77	1.27E-36	7.48	6.44E-27	0.81	2.86E-01
12	G__Lactobacillus	-9.77	1.65E-150	1.59	4.05E-02	8.28	2.08E-26	6.68	2.62E-17	0.93	3.08E-01

19	G__Lactobacillus	-8.95	2.92E-142	1.52	4.15E-02	7.67	2.39E-24	6.16	8.05E-16	0.98	2.37E-01
22	G__Lactobacillus	-9.21	9.15E-50	1.47	4.90E-02	7.58	7.20E-24	6.11	1.07E-15	0.86	3.24E-01
29	G__Lactobacillus	-8.84	4.89E-47	1.33	7.90E-02	6.90	5.74E-20	5.57	2.86E-13	0.91	2.73E-01
37	G__Lactobacillus	-8.30	1.95E-44	1.39	6.72E-02	6.45	1.70E-17	5.06	4.89E-11	0.89	3.17E-01
54	G__Lactobacillus	-7.31	1.02E-41	1.21	4.18E-02	5.48	1.50E-17	4.27	7.25E-11	0.53	4.58E-01
75	G__Streptococcus	-7.03	1.11E-40	0.78	3.04E-01	5.95	1.73E-17	5.17	2.19E-13	0.56	4.92E-01
115	G__Lactobacillus	-6.51	3.87E-40	1.28	7.20E-02	5.23	3.74E-13	3.95	8.43E-08	0.84	3.19E-01
127	G__Weissella	-6.21	2.26E-39	0.81	2.53E-01	5.32	5.19E-15	4.51	5.81E-11	0.33	7.49E-01
94	G__Streptococcus	-5.69	4.11E-39	0.88	2.33E-01	4.82	5.10E-12	3.95	2.99E-08	1.00	1.92E-01
136	G__Leuconostoc	-6.01	4.75E-38	0.70	4.33E-01	5.05	2.64E-11	4.35	1.43E-08	0.69	4.36E-01
170	G__Lactobacillus	-5.60	4.04E-32	0.46	6.12E-01	4.62	6.92E-11	4.16	5.95E-09	0.36	7.48E-01
72	G__Micrococcus	-2.70	1.11E-09	1.02	2.40E-01	3.99	2.04E-07	2.97	1.29E-04	1.07	2.11E-01
50	G__Staphylococcus	-3.80	1.88E-09	-0.97	3.33E-01	6.69	9.89E-15	7.66	7.78E-19	1.65	1.04E-01
255	G__Peptostreptococcus	-5.22	1.36E-31	1.27	1.04E-01	3.27	1.45E-05	2.00	1.09E-02	1.30	1.52E-01
204	G__Leuconostoc	-5.24	2.12E-26	0.85	2.73E-01	3.90	8.04E-08	3.05	3.81E-05	1.01	2.04E-01
272	G__Tepidimicrobium	-4.97	3.69E-26	1.30	7.90E-02	3.69	6.04E-07	2.39	1.91E-03	0.92	2.42E-01
214	G__Leuconostoc	-5.17	9.46E-25	0.90	2.29E-01	4.49	2.54E-10	3.59	6.48E-07	0.54	5.08E-01
221	G__Lactococcus	-4.80	5.17E-23	-0.89	2.71E-01	2.81	2.71E-04	3.71	7.70E-07	0.31	7.92E-01
254	G__Terrisporobacter	-5.23	5.63E-23	1.04	1.65E-01	3.14	1.69E-05	2.09	5.13E-03	0.66	4.15E-01
213	G__Lactobacillus	-5.03	1.71E-22	0.29	8.34E-01	3.92	2.49E-07	3.63	1.85E-06	1.06	1.95E-01
353	G__Bifidobacterium	-4.61	1.45E-21	0.44	6.59E-01	2.06	3.76E-03	1.62	2.54E-02	1.29	1.38E-01
367	G__Vagococcus	-4.66	8.95E-20	-0.09	1.00E+00	2.43	1.52E-03	2.53	8.77E-04	-0.10	9.83E-01
314	G__Lactobacillus	-5.00	9.45E-19	-0.18	9.45E-01	1.35	4.98E-02	1.52	2.10E-02	1.30	NA
290	G__Lactobacillus	-5.03	2.98E-18	0.70	3.60E-01	1.94	4.13E-03	1.25	6.91E-02	0.15	NA
329	G__Carnobacterium	-4.69	3.23E-17	-0.31	8.34E-01	2.72	5.99E-04	3.03	9.89E-05	0.01	NA
335	G__Carnobacterium	-4.83	3.36E-16	0.50	5.97E-01	2.23	1.82E-03	1.73	1.73E-02	0.23	8.92E-01
276	G__Lactobacillus	-4.67	5.52E-16	0.41	6.86E-01	3.76	3.10E-07	3.34	5.81E-06	0.86	2.79E-01
346	G__Geobacillus	-4.72	6.63E-15	2.51	1.81E-04	3.48	5.08E-07	0.97	1.97E-01	0.27	8.72E-01
321	G__Lactobacillus	-4.64	6.88E-15	0.28	8.38E-01	3.23	1.30E-05	2.95	7.13E-05	0.71	3.79E-01
361	G__Clostridium sensu stricto 18	-4.49	6.97E-15	0.48	6.14E-01	2.95	6.53E-05	2.47	9.01E-04	0.37	7.49E-01
142	G__Psychrobacter	-5.49	9.11E-15	-2.39	2.86E-03	2.03	1.93E-02	4.42	6.66E-08	0.20	9.42E-01
297	G__Vagococcus	-4.56	7.66E-14	1.19	9.44E-02	3.25	3.38E-06	2.06	4.92E-03	0.26	8.77E-01
88	G__Dermacoccus	-3.81	1.90E-13	0.54	5.15E-01	4.14	2.56E-09	3.59	3.03E-07	0.76	3.31E-01
444	G__Lactococcus	-4.01	2.62E-13	1.20	8.35E-02	2.72	7.48E-05	1.52	3.47E-02	0.84	2.65E-01
131	G__Allorhizobium	-3.09	2.33E-12	3.05	8.77E-05	2.82	2.10E-04	-0.23	8.04E-01	1.26	1.62E-01
379	G__Leuconostoc	-4.04	2.65E-12	0.82	3.09E-01	3.54	1.64E-06	2.72	2.85E-04	0.13	9.72E-01
403	G__Corynebacterium 1	-4.22	2.88E-12	0.05	1.00E+00	1.86	1.43E-02	1.82	1.46E-02	0.39	7.32E-01
380	G__Lactobacillus	-3.89	7.89E-12	0.98	2.65E-01	2.27	2.69E-03	1.30	9.42E-02	1.11	1.82E-01
266	G__Staphylococcus	-4.42	2.15E-11	2.35	4.72E-04	3.03	8.17E-06	0.68	3.66E-01	0.52	6.01E-01
499	G__Lactobacillus	-4.00	2.92E-11	0.03	1.00E+00	1.78	1.19E-02	1.75	1.18E-02	-0.26	8.69E-01
224	G__Micrococcus	-4.39	4.52E-11	2.35	6.79E-04	2.34	4.33E-04	-0.01	1.00E+00	-0.54	NA
441	G__Leuconostoc	-3.82	1.29E-10	1.58	1.84E-02	3.14	4.62E-06	1.56	3.18E-02	1.06	1.71E-01
445	G__Staphylococcus	-4.24	2.57E-10	-0.43	6.87E-01	1.58	3.65E-02	2.01	6.23E-03	0.29	8.69E-01
571	G__Gallicola	-3.82	5.84E-10	0.50	5.75E-01	1.82	7.43E-03	1.33	5.37E-02	0.37	NA

506	G__Lactobacillus	-3.66	8.46E-09	1.41	5.83E-02	2.90	7.74E-05	1.49	5.23E-02	1.09	1.81E-01
691	G__Terrisporobacter	-3.53	1.13E-08	1.75	3.60E-03	1.75	2.38E-03	0.00	1.00E+00	0.40	NA
141	G__Pseudomonas	-2.84	2.09E-07	1.71	1.08E-02	3.20	3.05E-06	1.49	3.57E-02	0.03	NA
205	F__Rhizobiaceae	-3.04	2.60E-07	0.57	4.92E-01	1.96	4.47E-03	1.38	4.66E-02	-0.22	NA
26	G__Streptococcus	-3.16	6.83E-07	0.59	5.34E-01	2.32	1.17E-03	1.72	1.58E-02	-0.12	9.76E-01
411	G__Acinetobacter	-3.02	3.29E-06	0.20	9.26E-01	1.46	3.35E-02	1.26	6.05E-02	-0.13	NA
303	G__Chryseobacterium	-3.00	4.88E-06	1.11	1.54E-01	1.56	2.73E-02	0.45	5.41E-01	0.08	9.96E-01
51	G__Cutibacterium	-2.42	6.49E-06	0.03	1.00E+00	2.21	1.06E-04	2.18	1.22E-04	0.38	6.66E-01
196	G__Corynebacterium 1	-2.83	1.12E-05	1.52	2.75E-02	2.84	5.26E-05	1.32	6.90E-02	-0.56	4.90E-01
260	G__Cutibacterium	-2.52	1.80E-04	1.46	2.86E-02	3.65	1.65E-07	2.18	2.56E-03	0.56	4.73E-01
61	G__Enhydrobacter	-2.17	2.72E-04	0.60	4.20E-01	4.46	2.46E-12	3.86	2.17E-09	0.65	3.74E-01
49	G__Staphylococcus	-2.37	4.49E-04	0.11	1.00E+00	3.46	1.08E-06	3.34	2.15E-06	0.31	7.83E-01
110	G__Staphylococcus	-2.65	4.52E-04	0.45	6.86E-01	2.49	1.01E-03	2.04	7.16E-03	0.96	2.37E-01
39	G__Verticia	-2.69	8.78E-04	-1.92	2.45E-02	1.98	2.66E-02	3.90	3.51E-06	-1.01	2.79E-01
81	G__Vibrionimonas	-1.94	1.42E-03	0.80	3.86E-01	2.81	1.57E-04	2.01	7.27E-03	0.18	9.42E-01
286	G__Acinetobacter	-2.62	6.31E-03	0.09	1.00E+00	2.10	3.76E-03	2.01	5.22E-03	0.34	7.49E-01
133	G__Variovorax	-2.30	7.21E-02	1.73	1.87E-02	2.58	3.30E-04	0.86	2.48E-01	0.83	3.21E-01
104	G__Rhodococcus	-1.89	3.67E-01	-0.69	4.47E-01	2.88	9.37E-05	3.57	7.77E-07	0.99	2.23E-01
299	G__Reyranella	-2.17	5.77E-01	3.46	1.07E-05	2.51	9.03E-04	-0.95	2.41E-01	0.50	6.80E-01
32	G__Mycoplasma	NA	NA	4.37	1.39E-06	8.07	1.77E-19	3.70	6.61E-05	1.48	1.69E-01
38	G__Mycoplasma	NA	NA	4.28	1.43E-06	8.03	1.13E-19	3.76	4.21E-05	1.27	2.19E-01
73	G__Deefgea	NA	NA	1.26	2.40E-01	7.47	1.35E-15	6.21	4.93E-11	-0.87	4.35E-01
67	G__Mycoplasma	NA	NA	4.27	9.99E-07	7.35	5.05E-17	3.08	7.69E-04	1.18	2.37E-01
124	G__Mycoplasma	NA	NA	4.71	6.72E-08	6.94	9.23E-15	2.23	1.83E-02	0.95	3.74E-01
113	G__Deefgea	NA	NA	1.54	1.16E-01	6.94	2.95E-14	5.40	5.93E-09	-1.02	3.50E-01
71	G__Deefgea	NA	NA	-0.16	1.00E+00	6.63	8.25E-14	6.79	2.26E-14	-0.07	9.96E-01
21	G__Verticia	NA	NA	-1.89	2.91E-02	2.38	6.47E-03	4.27	3.75E-07	0.74	4.58E-01
107	G__Mycoplasma	NA	NA	4.93	2.53E-08	6.54	1.44E-13	1.61	8.48E-02	1.42	1.72E-01
155	G__Deefgea	NA	NA	1.27	2.44E-01	6.17	1.03E-10	4.91	4.03E-07	-0.92	4.02E-01
116	G__Deefgea	NA	NA	0.18	1.00E+00	5.88	2.21E-10	5.70	8.54E-10	-0.23	9.34E-01
187	G__Deefgea	NA	NA	1.45	1.53E-01	5.69	1.09E-09	4.24	7.78E-06	-1.20	2.42E-01
169	G__Deefgea	NA	NA	0.37	8.44E-01	5.39	1.08E-08	5.02	1.15E-07	-1.75	1.23E-01
147	G__Deefgea	NA	NA	0.01	1.00E+00	5.30	3.51E-09	5.29	3.90E-09	-0.12	9.80E-01
249	G__Deefgea	NA	NA	0.70	5.39E-01	5.01	9.04E-09	4.31	9.63E-07	-1.68	7.08E-02
189	G__Deefgea	NA	NA	-0.33	8.53E-01	4.65	1.57E-07	4.99	1.58E-08	0.00	NA
215	F__Betaproteobacteriales Incertae	NA	NA	3.10	5.46E-05	4.45	1.01E-08	1.36	1.03E-01	0.69	4.58E-01
119	F__Enterobacteriaceae	NA	NA	1.12	2.53E-01	4.12	1.23E-06	2.99	4.73E-04	-0.01	NA
320	G__Aeromonas	NA	NA	-0.47	6.93E-01	4.07	1.02E-06	4.53	3.45E-08	-0.18	9.60E-01
358	G__Mycobacterium	NA	NA	0.54	6.01E-01	3.84	1.08E-06	3.29	3.46E-05	-0.96	2.57E-01
555	G__Methylobacterium	NA	NA	2.01	2.03E-03	3.66	1.10E-07	1.65	2.24E-02	0.37	7.21E-01
541	G__Methylobacterium	NA	NA	3.13	3.03E-07	3.64	5.36E-09	0.51	4.82E-01	0.38	6.94E-01
570	G__Methylobacterium	NA	NA	2.38	4.75E-05	3.64	3.18E-09	1.25	6.44E-02	0.88	2.10E-01
151	G__Buttiauxella	NA	NA	2.15	9.36E-03	3.51	2.45E-05	1.36	1.12E-01	0.67	4.92E-01
150	F__Enterobacteriaceae	NA	NA	0.84	4.69E-01	3.40	1.44E-04	2.56	4.56E-03	0.36	8.67E-01

175	G__Vibrionimonas	NA	NA	3.03	5.72E-07	3.37	1.36E-08	0.34	6.29E-01	-0.01	NA
112	G__Pseudomonas	NA	NA	0.16	9.96E-01	3.34	1.07E-05	3.18	2.52E-05	0.57	5.40E-01
248	G__Rudanella	NA	NA	-0.14	1.00E+00	3.13	1.01E-04	3.27	3.88E-05	0.37	7.49E-01
605	G__Staphylococcus	NA	NA	-0.72	4.97E-01	3.08	3.83E-04	3.80	7.23E-06	0.93	3.47E-01
446	G__Staphylococcus	NA	NA	-1.18	1.93E-01	3.05	4.33E-04	4.23	5.07E-07	1.16	2.13E-01
267	G__Rhodococcus	NA	NA	-0.19	9.65E-01	2.98	1.05E-04	3.16	3.09E-05	0.17	9.44E-01
438	G__Bacillus	NA	NA	1.61	1.98E-02	2.97	2.46E-05	1.36	6.57E-02	-0.54	5.53E-01
706	G__Clostridium sensu stricto 7	NA	NA	1.80	1.11E-02	2.90	4.93E-05	1.10	1.46E-01	0.66	4.24E-01
738	G__Methylobacterium	NA	NA	1.48	2.84E-02	2.86	3.60E-05	1.38	5.46E-02	-0.06	9.96E-01
637	G__Vagococcus	NA	NA	2.22	8.04E-04	2.78	3.31E-05	0.56	4.54E-01	-0.32	7.54E-01
534	G__Aeromonas	NA	NA	-0.36	7.76E-01	2.72	5.22E-04	3.08	6.54E-05	-0.19	9.17E-01
191	G__Haemophilus	NA	NA	2.08	1.73E-03	2.59	1.02E-04	0.51	4.92E-01	0.62	4.37E-01
492	G__Bradyrhizobium	NA	NA	3.13	2.05E-05	2.57	2.58E-04	-0.56	4.69E-01	1.27	1.59E-01
366	G__Variovorax	NA	NA	0.92	2.58E-01	2.45	9.35E-04	1.52	4.58E-02	0.79	3.63E-01
565	G__Lactobacillus	NA	NA	1.52	3.15E-02	2.39	6.65E-04	0.87	2.41E-01	1.34	9.24E-02
171	G__Janthinobacterium	NA	NA	-0.13	1.00E+00	2.39	2.06E-03	2.52	1.04E-03	0.01	NA
539	G__Reyranella	NA	NA	2.12	6.17E-03	2.35	1.88E-03	0.23	8.04E-01	0.61	5.08E-01
185	G__Rhodococcus	NA	NA	0.34	7.75E-01	2.33	1.69E-03	1.98	7.75E-03	-0.54	5.95E-01
250	F__Burkholderiaceae	NA	NA	2.27	6.38E-04	2.27	3.72E-04	0.00	1.00E+00	0.26	NA
360	G__Rhodococcus	NA	NA	0.30	8.34E-01	2.24	3.11E-03	1.94	1.21E-02	1.24	1.62E-01
542	F__Betaproteobacteriales	NA	NA	2.14	1.49E-03	2.13	9.62E-04	-0.01	1.00E+00	-0.43	NA
513	G__Micrococcus	NA	NA	0.89	2.67E-01	2.09	3.65E-03	1.20	1.05E-01	0.58	NA
461	G__Acinetobacter	NA	NA	2.17	3.17E-03	2.08	2.96E-03	-0.09	9.48E-01	-0.87	2.73E-01
654	G__Candidatus Megaira	NA	NA	2.07	1.87E-03	2.07	1.19E-03	0.00	1.00E+00	0.38	NA
103	G__Buttiauxella	NA	NA	-0.16	1.00E+00	2.07	1.36E-02	2.23	7.55E-03	-1.18	2.08E-01
726	G__Clostridium sensu stricto 18	NA	NA	0.64	4.20E-01	2.06	2.41E-03	1.42	4.13E-02	0.61	4.36E-01
342	G__Pseudomonas	NA	NA	1.97	3.04E-03	1.97	1.93E-03	0.00	1.00E+00	0.50	NA
373	G__Hydrogenophaga	NA	NA	1.45	2.91E-02	1.96	2.70E-03	0.50	4.81E-01	-0.19	NA
111	G__Delftia	NA	NA	0.65	5.02E-01	1.94	9.04E-03	1.29	8.01E-02	0.29	8.69E-01
528	G__Phreatobacter	NA	NA	-1.90	3.04E-03	1.92	8.04E-03	3.82	2.18E-08	-0.63	4.10E-01
689	G__Bradyrhizobium	NA	NA	0.53	5.45E-01	1.88	7.10E-03	1.35	5.43E-02	-0.63	NA
631	G__Brevinema	NA	NA	-0.15	1.00E+00	1.85	1.21E-02	2.00	6.23E-03	1.33	1.31E-01
620	G__Lactobacillus	NA	NA	0.27	8.34E-01	1.80	1.04E-02	1.53	2.41E-02	-1.04	1.71E-01
650	G__Lactobacillus	NA	NA	0.23	8.90E-01	1.78	1.08E-02	1.55	2.77E-02	1.36	6.23E-02
53	G__Streptococcus	NA	NA	1.84	3.20E-03	1.73	3.56E-03	-0.11	9.13E-01	0.28	NA
100	G__Allorhizobium	NA	NA	2.48	1.02E-03	1.68	2.21E-02	-0.80	2.68E-01	0.04	NA
572	F__Solimonadaceae	NA	NA	1.06	1.10E-01	1.46	1.93E-02	0.41	5.41E-01	1.24	NA
812	G__Acinetobacter	NA	NA	0.73	3.01E-01	1.46	2.08E-02	0.73	2.65E-01	-0.94	NA
546	G__Phreatobacter	NA	NA	0.30	8.05E-01	1.43	3.89E-02	1.13	9.79E-02	1.69	NA
386	G__Sphingomonas	NA	NA	0.88	NA	1.39	2.28E-02	0.50	4.41E-01	-0.28	NA
621	G__Aeromonas	NA		-1.19	1.01E-01	2.13	4.17E-03	3.32	3.72E-06	-0.58	4.73E-01
704	G__Nevskia	NA		1.03	1.16E-01	2.10	1.26E-03	1.07	1.12E-01	-0.56	NA
149	G__Aeromonas	NA	NA	-6.57	2.28E-17	-0.01	1.00E+00	6.56	3.27E-18	-0.44	7.24E-01
52	G__Aeromonas	NA	NA	-6.45	9.55E-15	0.04	1.00E+00	6.49	1.95E-15	-0.48	6.95E-01

208	O__Actinomycetales	NA	NA	-6.20	1.28E-15	0.00	1.00E+00	6.20	2.39E-16	-0.09	9.94E-01
76	G__Aeromonas	NA	NA	-6.19	9.95E-14	-0.07	1.00E+00	6.12	6.02E-14	-0.52	6.88E-01
36	G__Aeromonas	NA	NA	-6.02	4.32E-10	-0.83	4.65E-01	5.19	3.43E-08	0.25	9.17E-01
238	G__Aeromonas	NA	NA	-5.92	4.31E-15	-0.01	1.00E+00	5.92	1.05E-15	-0.41	7.49E-01
140	G__Aeromonas	NA	NA	-5.73	5.59E-13	-0.24	9.12E-01	5.49	1.44E-12	-0.62	6.12E-01
274	G__Psychrobacter	NA	NA	-5.34	4.70E-11	0.01	1.00E+00	5.35	1.93E-11	0.79	4.05E-01
383	G__Psychrobacter	NA	NA	-5.26	4.65E-11	0.00	1.00E+00	5.26	1.88E-11	0.06	9.96E-01
79	G__Aeromonas	NA	NA	-5.24	1.37E-09	0.21	9.54E-01	5.45	1.14E-10	-0.53	6.94E-01
135	G__Crenobacter	NA	NA	-4.70	7.44E-09	-0.29	8.68E-01	4.41	1.77E-08	-0.81	3.88E-01
573	G__Aeromonas	NA	NA	-3.98	4.98E-09	-0.01	1.00E+00	3.97	2.22E-09	-0.85	2.76E-01
616	F__Chromobacteriaceae	NA		-3.91	4.98E-09	-0.01	1.00E+00	3.90	2.17E-09	-1.05	1.74E-01
676	G__Shinella	NA	NA	-3.88	6.74E-08	-0.02	1.00E+00	3.87	3.00E-08	-1.03	2.19E-01
711	G__Pseudomonas	NA		-3.75	3.59E-08	-0.01	1.00E+00	3.75	1.60E-08	-0.59	4.66E-01
556	G__Streptococcus	NA	NA	-3.72	5.72E-07	0.41	7.35E-01	4.13	2.46E-08	-0.08	9.96E-01
157	F__Enterobacteriaceae	NA	NA	-3.40	5.56E-06	-1.04	2.15E-01	2.35	5.21E-04	-0.25	8.88E-01
634	G__Chryseobacterium	NA	NA	-3.25	2.86E-06	0.00	1.00E+00	3.25	1.28E-06	-0.05	9.96E-01
192	G__Rickettsiella	NA	NA	-3.23	7.57E-07	-0.01	1.00E+00	3.22	3.33E-07	-0.64	3.98E-01
87	G__Burkholderia	NA	NA	-3.22	8.59E-06	-0.01	1.00E+00	3.21	3.85E-06	-0.66	4.41E-01
437	G__Pseudomonas	NA	NA	-3.22	3.11E-06	0.91	2.85E-01	4.13	5.53E-09	-0.11	9.76E-01
44	G__Verticia	-2.86	1.36E-04	-3.22	1.49E-05	0.30	8.51E-01	3.51	1.47E-06	0.06	9.96E-01
287	F__Pirellulaceae	NA		-2.80	6.03E-05	0.00	1.00E+00	2.80	2.79E-05	0.14	9.69E-01
245	G__Pseudomonas	NA	NA	-2.77	9.76E-05	0.58	5.24E-01	3.35	1.62E-06	0.51	6.50E-01
458	G__Undibacterium	NA	NA	-2.77	5.11E-05	-0.01	1.00E+00	2.76	2.42E-05	-0.68	3.82E-01
132	G__Rickettsiella	NA	NA	-2.72	4.75E-05	-0.02	1.00E+00	2.70	2.31E-05	-1.25	1.36E-01
581	F__Enterobacteriaceae	NA	NA	-2.66	1.81E-04	0.56	5.57E-01	3.21	5.29E-06	-0.15	9.69E-01
781	G__Pseudomonas	NA	NA	-2.53	4.76E-04	1.14	1.79E-01	3.66	4.82E-07	-0.89	2.76E-01
45	G__Verticia	-2.08	6.86E-01	-2.51	5.43E-04	0.88	3.13E-01	3.38	3.03E-06	0.57	5.95E-01
234	G__Psychrobacter	NA	NA	-2.50	1.86E-04	0.56	5.34E-01	3.06	5.11E-06	-0.24	8.88E-01
476	G__Psychrobacter	NA	NA	-2.50	3.05E-04	-0.01	1.00E+00	2.49	1.65E-04	-0.77	3.48E-01
829	G__Defluviimonas	NA		-2.30	6.87E-04	-0.01	1.00E+00	2.29	4.02E-04	-0.90	2.37E-01
247	G__Acinetobacter	NA	NA	-2.23	5.16E-03	1.45	8.98E-02	3.68	3.08E-06	-0.05	9.96E-01
558	G__Pseudomonas	NA	NA	-2.19	1.15E-03	0.00	1.00E+00	2.19	6.66E-04	-0.27	NA
568	G__Pseudorhodobacter	NA	NA	-2.17	9.50E-04	0.91	2.46E-01	3.08	3.92E-06	-0.49	6.44E-01
451	G__Psychrobacter	NA	NA	-2.09	1.70E-03	0.22	9.02E-01	2.30	3.83E-04	-0.79	NA
690	G__Brevibacterium	NA	NA	-2.08	1.99E-03	0.00	1.00E+00	2.09	1.14E-03	0.58	NA
481	G__Pseudomonas	NA	NA	-2.07	3.49E-03	1.06	2.03E-01	3.12	1.20E-05	0.53	6.47E-01
180	G__Rickettsiella	NA	NA	-2.04	3.11E-03	-0.17	9.54E-01	1.87	3.79E-03	-1.27	1.21E-01
464	G__Flavobacterium	NA	NA	-2.04	3.24E-03	-0.72	3.47E-01	1.31	3.35E-02	-0.06	9.96E-01
615	G__Acinetobacter	NA	NA	-1.87	8.12E-03	0.75	3.67E-01	2.62	1.85E-04	-0.46	6.66E-01
821	G__Flavobacterium	NA		-1.86	6.40E-03	0.54	5.49E-01	2.40	3.83E-04	-0.82	NA
284	G__Rickettsiella	NA	NA	-1.78	1.09E-02	-0.01	1.00E+00	1.77	7.13E-03	-1.19	NA
658	G__Acinetobacter	NA	NA	-1.74	1.25E-02	0.85	2.95E-01	2.59	1.88E-04	-0.28	8.15E-01
582	G__Aeromonas	NA	NA	-1.64	1.22E-02	0.43	6.18E-01	2.07	1.12E-03	-1.24	8.88E-02
477	O__Betaproteobacteriales	NA	NA	-1.57	1.82E-02	0.01	1.00E+00	1.58	1.07E-02	0.76	NA

391	F__Enterobacteriaceae	NA	NA	-1.54	3.35E-02	-0.81	2.78E-01	0.73	2.41E-01	0.48	NA
159	G__Exiguobacterium	NA	NA	-1.51	3.57E-02	1.01	2.14E-01	2.52	3.77E-04	0.22	9.04E-01
295	G__Brevundimonas	NA	NA	-1.50	4.18E-02	0.75	3.67E-01	2.24	1.59E-03	-0.05	9.96E-01
336	G__DSSD61	NA	NA	-1.47	2.91E-02	0.01	1.00E+00	1.47	1.75E-02	0.87	NA
356	G__Stenotrophomonas	NA	NA	-1.46	3.96E-02	1.16	1.42E-01	2.62	2.29E-04	0.12	9.75E-01
270	G__Bradyrhizobium	NA	NA	2.02	1.87E-02	1.57	5.53E-02	-0.45	5.96E-01	1.03	2.42E-01
86	G__Allorhizobium	-2.70	2.23E-08	2.29	1.19E-02	1.30	1.53E-01	-0.99	2.48E-01	0.90	3.74E-01
4	F__Mycoplasmataceae	-3.03	8.73E-20	2.81	1.92E-04	0.93	2.38E-01	-1.88	9.82E-03	-0.92	2.62E-01
6	F__Mycoplasmataceae	-3.05	3.82E-15	2.94	2.62E-04	0.79	3.63E-01	-2.15	5.54E-03	-1.02	2.37E-01
1	F__Mycoplasmataceae	-2.93	2.16E-18	2.95	6.88E-05	1.29	8.26E-02	-1.66	2.02E-02	0.22	9.06E-01
24	F__Mycoplasmataceae	-3.15	1.31E-11	3.03	7.54E-04	-0.01	1.00E+00	-3.04	4.02E-04	-0.96	3.63E-01
183	G__Tardiphaga	-2.86	2.88E-05	3.29	9.45E-05	0.71	4.28E-01	-2.58	1.58E-03	0.82	3.76E-01
241	G__Rhodoferax	NA	NA	1.37	7.90E-02	-0.20	8.80E-01	-1.57	2.10E-02	-0.58	NA
562	G__Roseiarcus	NA	NA	0.62	5.02E-01	-0.87	1.84E-01	-1.50	2.02E-02	0.23	NA
680	G__Brochothrix	-3.41	1.15E-08	-1.12	NA	0.17	9.46E-01	1.29	3.58E-02	0.96	NA
442	G__Citrobacter	NA	NA	-1.36	NA	-0.01	1.00E+00	1.35	3.78E-02	-0.91	NA
795	G__Ezakiella	NA	NA	-1.39	NA	0.00	1.00E+00	1.39	1.83E-02	0.21	NA
693	G__Rudanella	NA		-0.38	6.68E-01	1.03	1.39E-01	1.41	2.72E-02	-0.32	NA
694	G__Phreatobacter	NA	NA	-1.54	NA	-0.01	1.00E+00	1.53	1.00E-02	-0.58	NA
85	G__Rhodococcus	-2.67	2.93E-06	-0.69	4.79E-01	0.86	2.85E-01	1.55	3.28E-02	0.49	6.66E-01
707	G__Staphylococcus	NA	NA	-1.30	6.57E-02	0.27	8.28E-01	1.57	1.70E-02	-1.25	NA
212	G__Staphylococcus	NA	NA	-0.41	6.63E-01	1.22	9.17E-02	1.63	1.49E-02	1.05	NA
563	G__Lactobacillus	-3.93	3.71E-10	-0.39	6.69E-01	1.25	6.12E-02	1.64	1.23E-02	1.30	NA
564	G__Massilia	NA	NA	-1.02	1.50E-01	0.68	3.89E-01	1.70	9.76E-03	0.52	NA
824	O__Bacillales	NA	NA	-1.79	NA	0.00	1.00E+00	1.79	3.67E-03	0.14	NA
414	G__Glutamicibacter	-4.31	1.69E-11	-1.27	5.72E-02	0.54	5.17E-01	1.80	4.99E-03	-0.81	NA
9	F__Rickettsiaceae	-6.45	1.06E-28	-0.79	4.59E-01	1.09	2.15E-01	1.88	1.96E-02	1.11	2.19E-01
709	G__Flavobacterium	NA	NA	-1.04	1.52E-01	0.85	2.73E-01	1.89	5.30E-03	-0.06	NA
268	G__Lawsonella	NA	NA	-0.71	3.57E-01	1.19	1.00E-01	1.90	5.16E-03	0.48	NA
152	F__Paracaedibacteraceae	NA	NA	-1.17	1.01E-01	1.06	1.68E-01	2.23	1.22E-03	0.03	NA
126	G__Chryseobacterium	NA	NA	-1.16	1.28E-01	1.11	1.59E-01	2.27	1.19E-03	1.01	2.06E-01
92	F__Clostridiaceae 1	5.25	5.58E-12	-0.54	5.97E-01	-1.30	5.63E-02	-0.76	2.41E-01	0.80	NA
123	F__Holosporaceae	3.48	9.24E-06	-0.42	7.73E-01	-0.39	7.35E-01	0.02	1.00E+00	-0.91	3.27E-01
68	G__Carnobacterium	3.01	2.73E-05	-0.92	4.08E-01	-0.84	3.83E-01	0.08	9.70E-01	0.88	3.75E-01
398	G__Lactobacillus	-4.46	2.28E-13	-0.21	NA	0.75	2.85E-01	0.95	1.31E-01	1.12	NA
243	G__Carnobacterium	-5.49	3.70E-14	-0.53	NA	0.60	4.12E-01	1.14	6.91E-02	-0.36	NA
80	F__Erysipelotrichaceae	7.82	2.73E-29	0.00	NA	0.00	NA	0.00	NA	0.00	NA
118	F__Erysipelotrichaceae	7.18	4.64E-25	0.00	NA	0.00	NA	0.00	NA	0.00	NA
120	F__Erysipelotrichaceae	7.17	4.61E-26	0.00	NA	0.00	NA	0.00	NA	0.00	NA
158	F__Erysipelotrichaceae	6.67	1.33E-21	0.00	NA	-0.47	NA	-0.47	NA	0.31	NA
43	F__Clostridiaceae 1	6.49	1.08E-15	0.00	NA	0.00	NA	0.00	NA	0.00	NA
186	O__Micrococcales	6.24	6.18E-14	NA	NA	NA	NA	NA	NA	NA	NA
106	G__Yersinia	5.63	1.24E-22	NA	NA	NA	NA	NA	NA	NA	NA
70	G__Yersinia	5.62	1.24E-22	0.00	NA	-0.99	NA	-0.99	NA	0.06	NA

82	F__Clostridiaceae 1	5.61	4.10E-13	0.00	NA	-0.43	NA	-0.43	NA	0.28	NA
279	F__Mycoplasmataceae	5.52	2.00E-15	0.00	NA	-0.27	NA	-0.27	NA	0.18	NA
130	G__Yersinia	5.31	1.16E-19	-0.16	NA	-0.42	NA	-0.27	NA	0.39	NA
114	G__Hafnia-Obesumbacterium	4.99	2.79E-10	0.00	NA	-0.56	NA	-0.56	NA	-0.37	NA
487	F__Microbacteriaceae	4.63	1.87E-11	NA	NA	NA	NA	NA	NA	NA	NA
174	G__Hafnia-Obesumbacterium	4.38	6.29E-10	NA	NA	NA	NA	NA	NA	NA	NA
42	G__Yersinia	4.33	2.40E-12	0.00	NA	0.08	NA	0.08	NA	-0.06	NA
164	G__Hafnia-Obesumbacterium	4.26	4.91E-08	NA	NA	NA	NA	NA	NA	NA	NA
594	G__Chthoniobacter	4.10	2.04E-11	0.00	NA	-0.45	NA	-0.45	NA	-0.30	NA
427	G__Rhodobacter	4.05	2.13E-14	0.00	NA	0.00	NA	0.00	NA	0.00	NA
462	G__Nocardioides	4.03	1.28E-10	0.00	NA	-0.85	NA	-0.85	NA	0.05	NA
611	F__Mycoplasmataceae	3.97	6.40E-09	0.00	NA	-0.65	NA	-0.65	NA	-0.44	NA
552	G__Rickettsiella	3.94	5.38E-11	NA	NA	NA	NA	NA	NA	NA	NA
502	F__Rhizobiaceae	3.93	2.56E-11	0.24	NA	-0.67	NA	-0.91	NA	-0.45	NA
567	G__Paeniclostridium	3.87	2.56E-11	0.00	NA	-0.59	NA	-0.59	NA	0.39	NA
178	G__Ignatzschineria	3.82	2.99E-06	0.00	NA	-0.33	NA	-0.33	NA	-0.22	NA
577	F__Halieaceae	3.77	3.55E-10	0.00	NA	-0.33	NA	-0.33	NA	0.21	NA
721	G__Pirellula	3.73	8.81E-12	0.00	NA	-1.08	NA	-1.08	NA	0.05	NA
588	C__Parcubacteria	3.72	2.58E-10	0.00	NA	-0.39	NA	-0.39	NA	-0.26	NA
783	F__Microbacteriaceae	3.71	1.78E-07	NA	NA	NA	NA	NA	NA	NA	NA
779	F__Mycoplasmataceae	3.68	1.96E-07	NA	NA	NA	NA	NA	NA	NA	NA
282	G__Hafnia-Obesumbacterium	3.68	5.31E-07	NA	NA	NA	NA	NA	NA	NA	NA
730	G__Romboutsia	3.64	2.22E-07	NA	NA	NA	NA	NA	NA	NA	NA
530	O__Betaproteobacteriales	3.64	4.79E-09	-0.16	NA	-0.96	NA	-0.80	NA	-0.75	NA
465	O__Saccharimonadales	3.63	8.45E-09	0.00	NA	-0.66	NA	-0.67	NA	0.44	NA
667	G__Aeromonas	3.59	1.90E-07	0.00	NA	0.00	NA	0.00	NA	0.00	NA
355	O__PeM15	3.58	6.82E-09	0.09	NA	-0.29	NA	-0.38	NA	0.30	NA
540	G__Iamia	3.58	1.24E-10	-0.30	NA	-0.85	NA	-0.55	NA	0.36	NA
235	G__Hafnia-Obesumbacterium	3.57	6.49E-06	NA	NA	NA	NA	NA	NA	NA	NA
580	G__Dinghuibacter	3.56	2.78E-07	0.30	NA	-0.61	NA	-0.91	NA	-0.41	NA
675	F__Peptostreptococcaceae	3.49	8.47E-08	0.00	NA	-0.93	NA	-0.94	NA	-0.63	NA
278	G__Hafnia-Obesumbacterium	3.49	2.50E-05	NA	NA	NA	NA	NA	NA	NA	NA
787	G__Chthoniobacter	3.46	4.17E-08	0.00	NA	-0.73	NA	-0.73	NA	0.48	NA
323	G__Clostridium sensu stricto 1	3.46	1.39E-08	0.00	NA	-0.96	NA	-0.96	NA	-0.65	NA
834	O__Rhizobiales	3.46	3.55E-08	-0.20	NA	-0.56	NA	-0.36	NA	-0.57	NA
365	G__Bacillus	3.36	1.08E-09	0.00	NA	-1.02	NA	-1.02	NA	0.67	NA
748	G__Candidatus Udaeobacter	3.18	1.61E-07	0.00	NA	-0.76	NA	-0.76	NA	0.50	NA
372	G__Aeromonas	3.00	8.35E-05	0.56	NA	0.17	NA	-0.39	NA	-0.12	NA
586	F__Beijerinckiaceae	2.88	3.49E-06	0.00	NA	-1.35	NA	-1.35	NA	-0.30	NA
660	G__Luteolibacter	2.72	2.84E-04	NA	NA	NA	NA	NA	NA	NA	NA
688	G__Rhodobacter	2.68	1.49E-04	-0.75	NA	-0.48	NA	0.27	NA	-0.19	NA
509	G__Bacillus	2.66	1.56E-03	-0.84	NA	-0.01	NA	0.83	NA	-0.56	NA
759	G__Aeromonas	2.55	4.03E-03	0.00	NA	0.00	NA	0.00	NA	0.00	NA
678	F__Halieaceae	2.52	1.16E-03	0.00	NA	-0.99	NA	-0.99	NA	-0.67	NA

719	G__Pirellula	2.33	1.03E-02	0.00	NA	-0.94	NA	-0.94	NA	0.20	NA
148	G__Carnobacterium	-6.47	2.62E-23	-0.92	NA	-0.01	NA	0.92	NA	-0.62	NA
305	G__Enterococcus	-5.30	7.60E-15	NA	NA	NA	NA	NA	NA	NA	NA
253	G__Psychrobacter	-5.22	2.39E-13	0.20	NA	0.55	NA	0.35	NA	0.60	NA
322	G__Lactobacillus	-5.14	4.37E-13	NA	NA	NA	NA	NA	NA	NA	NA
354	G__Psychrobacter	-4.63	2.54E-12	0.00	NA	0.00	NA	0.00	NA	0.00	NA
324	G__Psychrobacter	-4.62	2.54E-11	0.23	NA	0.62	NA	0.40	NA	-0.69	NA
397	G__Lactobacillus	-4.54	2.76E-13	-0.78	NA	0.00	NA	0.78	NA	0.52	NA
467	G__Lactobacillus	-4.32	1.74E-10	NA	NA	NA	NA	NA	NA	NA	NA
521	G__Brevibacterium	-4.27	6.50E-10	NA	NA	NA	NA	NA	NA	NA	NA
179	G__Legionella	-4.15	3.92E-09	NA	NA	NA	NA	NA	NA	NA	NA
535	G__Lactobacillus	-4.08	1.24E-09	0.00	NA	0.00	NA	0.00	NA	0.00	NA
507	G__Lactobacillus	-4.06	2.25E-10	NA	NA	NA	NA	NA	NA	NA	NA
531	Unclassified bacteria	-3.93	1.11E-07	NA	NA	NA	NA	NA	NA	NA	NA
569	G__Pseudochrobactrum	-3.85	6.42E-10	-1.00	NA	-0.01	NA	0.99	NA	-0.67	NA
277	G__Limnohabitans	-3.77	2.27E-08	0.16	NA	0.16	NA	0.00	NA	0.11	NA
701	G__Vagococcus	-3.74	3.44E-09	NA	NA	NA	NA	NA	NA	NA	NA
301	G__Pseudomonas	-3.62	2.29E-08	-1.15	NA	0.01	1.00E+00	1.16	5.32E-02	0.77	NA
457	G__Pseudomonas	-3.60	3.58E-08	-0.07	NA	0.68	NA	0.75	NA	-0.05	NA
325	G__Micrococcus	-3.50	5.05E-08	-0.98	NA	0.00	NA	0.98	NA	0.65	NA
328	Unclassified bacteria	-3.49	6.14E-06	NA	NA	NA	NA	NA	NA	NA	NA
629	F__Amoebophilaceae	-3.47	2.91E-06	NA	NA	NA	NA	NA	NA	NA	NA
770	G__Lactobacillus	-3.45	8.29E-07	0.64	NA	0.64	NA	0.00	NA	0.43	NA
422	G__Alkanindiges	-3.41	5.04E-07	NA	NA	NA	NA	NA	NA	NA	NA
294	G__Sphaerotilus	-3.35	7.03E-06	NA	NA	NA	NA	NA	NA	NA	NA
318	G__Flavobacterium	-3.22	7.11E-06	-0.80	NA	-0.55	NA	0.25	NA	-0.90	NA
413	Unclassified bacteria	-2.58	1.09E-04	-0.57	NA	0.25	NA	0.82	NA	0.29	NA

Table S3. Differentially abundant skin OTUs

ASV no.	Taxonomy	Pre-translocation Wild:Hatchery		Treatment Hatchery:Enriched		Treatment Hatchery:Natural		Treatment Enriched:Natural		Origin Hatchery:Wild	
		Log2 FC	FDR	Log2 FC	FDR	Log2 FC	FDR	Log2 FC	FDR	Log2 FC	FDR
9	F__Rickettsiaceae	-9.75	1.22E-125	0.26	1.00E+00	3.16	2.43E-09	2.90	8.89E-08	2.11	4.44E-03
31	F__Rickettsiaceae	-8.28	2.20E-96	0.27	1.00E+00	4.38	5.09E-17	4.11	7.37E-15	2.06	4.09E-03
23	F__Rickettsiaceae	-8.56	1.37E-99	0.37	8.61E-01	3.49	5.96E-12	3.12	8.57E-10	2.06	4.36E-03
48	F__Rickettsiaceae	-7.56	3.23E-76	-0.02	1.00E+00	3.72	2.95E-11	3.75	1.89E-11	2.29	2.54E-03
30	F__Rickettsiaceae	-8.52	3.33E-97	0.06	1.00E+00	2.61	1.31E-05	2.55	9.43E-05	2.02	5.82E-03
27	F__Rickettsiaceae	-8.49	4.41E-97	-0.30	1.00E+00	2.41	3.96E-04	2.70	6.10E-06	2.33	2.70E-03
174	G__Hafnia-Obesumbacterium	1.15	NA	2.29	9.91E-03	2.30	1.30E-02	0.01	NA	4.48	5.32E-05
745	F__Burkholderiaceae	0.99	NA	1.26	3.77E-01	2.49	2.45E-02	1.23	5.90E-01	5.74	1.43E-05
1077	G__Sphingobacterium	NA	NA	0.31	1.00E+00	2.09	3.65E-02	1.78	1.58E-01	4.09	3.51E-04
4	F__Mycoplasmataceae	0.23	8.79E-01	0.62	9.75E-01	-3.00	1.28E-02	-3.62	2.66E-03	4.37	3.89E-02
360	G__Rhodococcus	-0.58	6.35E-01	0.03	1.00E+00	-2.28	3.19E-02	-2.32	4.42E-02	3.87	1.52E-02
760	G__Pseudomonas	-3.28	9.92E-06	-1.28	3.44E-01	2.02	9.16E-02	3.30	2.52E-03	3.93	6.37E-03
73	G__Deefgea	0.52	NA	1.61	8.18E-02	1.42	1.31E-01	-0.18	NA	2.78	2.53E-02
324	G__Psychrobacter	NA	NA	-0.40	1.00E+00	1.41	2.20E-01	1.81	1.51E-01	3.71	6.18E-03
477	O__Betaproteobacteriales	-0.62	NA	-1.44	2.13E-01	1.56	2.33E-01	3.00	9.36E-03	3.07	4.57E-02
1119	G__Comamonas	NA	NA	-0.84	5.26E-01	1.15	3.97E-01	1.98	1.02E-01	3.55	7.15E-03
344	G__Pseudomonas	-2.55	4.11E-03	-1.28	3.79E-01	1.20	5.21E-01	2.49	4.52E-02	4.47	6.03E-03
614	G__Pseudomonas	-2.78	3.41E-04	-0.39	1.00E+00	0.62	8.86E-01	1.01	7.70E-01	3.89	7.45E-03
758	F__Solimonadaceae	-2.02	5.40E-03	1.97	8.74E-02	-2.00	5.53E-02	-3.97	2.53E-06	-3.86	6.27E-03
448	G__DSSD61	-1.78	7.46E-02	2.71	1.97E-02	-1.20	3.91E-01	-3.91	1.38E-05	-5.28	1.78E-04
200	F__Solimonadaceae	-0.61	6.18E-01	2.36	9.83E-03	-1.44	1.40E-01	-3.80	8.47E-07	-4.39	2.19E-04
572	F__Solimonadaceae	-1.11	NA	2.46	9.08E-03	-1.28	1.89E-01	-3.73	1.29E-06	-4.81	5.32E-05
227	G__Pelomonas	-1.33	1.87E-01	1.39	1.72E-01	-2.26	8.16E-03	-3.65	3.75E-08	-6.09	1.40E-07
741	G__Aquabacterium	-1.95	NA	2.24	5.75E-02	-1.15	4.08E-01	-3.40	2.45E-03	-4.37	1.97E-03
336	G__DSSD61	-0.47	6.94E-01	1.03	5.38E-01	-1.90	1.01E-01	-2.94	1.47E-02	-5.25	4.17E-04
674	G__Pelomonas	-0.69	NA	0.73	7.61E-01	-1.75	1.09E-01	-2.48	3.17E-02	-4.18	6.18E-03
671	G__Sediminibacterium	-1.89	1.02E-02	0.60	8.67E-01	-1.76	1.16E-01	-2.35	4.48E-02	-3.78	3.66E-02
642	G__Undibacterium	1.94	NA	-2.01	5.66E-02	1.51	2.46E-01	3.52	6.69E-04	-3.02	4.57E-02
849	F__Enterobacteriaceae	0.00	NA	-1.58	1.78E-01	2.39	5.53E-02	3.98	1.13E-04	-4.76	1.04E-03
546	G__Phreatobacter	-0.12	NA	1.05	5.66E-01	-0.64	8.86E-01	-1.69	3.57E-01	-5.21	7.02E-04
239	G__Yersinia	0.00	NA	1.21	3.74E-01	0.13	NA	-1.07	6.82E-01	-3.38	1.61E-02
657	G__Diaphorobacter	-2.69	NA	-0.76	7.98E-01	-1.26	4.62E-01	-0.50	NA	-4.56	8.86E-03
268	G__Lawsonella	1.89	1.06E-02	-1.60	2.37E-01	-1.41	3.81E-01	0.19	NA	3.41	5.10E-02
99	F__Burkholderiaceae	-0.06	NA	0.00	1.00E+00	-8.84	2.97E-40	-8.84	2.96E-40	0.00	1.00E+00
166	F__Burkholderiaceae	NA	NA	0.00	NA	-7.86	1.09E-32	-7.86	1.63E-32	0.00	1.00E+00
190	F__Burkholderiaceae	NA	NA	0.00	NA	-7.63	1.02E-28	-7.63	1.36E-28	0.00	1.00E+00
232	G__Limnohabitans	NA	NA	0.00	NA	-7.18	1.35E-28	-7.18	1.68E-28	0.00	1.00E+00
302	F__Burkholderiaceae	NA	NA	0.00	NA	-6.87	8.26E-26	-6.87	9.87E-26	0.00	1.00E+00

806	G__Pirellula	NA	NA	0.00	NA	-4.92	2.92E-14	-4.92	3.17E-14	0.00	1.00E+00
454	G__Acidovorax	NA	NA	0.74	7.40E-01	-4.50	2.07E-09	-5.24	2.04E-12	-2.83	5.58E-02
769	G__CM1G08	0.34	NA	0.00	1.00E+00	-4.69	1.05E-11	-4.69	1.04E-11	0.00	1.00E+00
1080	G__Alkanindiges	NA	NA	0.00	NA	-4.37	8.19E-11	-4.37	7.31E-11	0.00	1.00E+00
597	G__Rhodoferax	2.00	NA	0.00	1.00E+00	-4.71	2.02E-10	-4.71	1.92E-10	0.00	1.00E+00
958	G__Dyadobacter	-0.84	NA	0.00	1.00E+00	-4.48	2.47E-10	-4.48	2.24E-10	0.00	1.00E+00
942	G__Spirosoma	NA	NA	0.00	NA	-4.51	2.57E-10	-4.51	2.31E-10	0.00	1.00E+00
519	G__Sphingomonas	-0.53	NA	0.74	6.82E-01	-3.81	6.96E-08	-4.56	2.31E-10	-1.46	5.84E-01
1128	F__Solimonadaceae	NA	NA	0.00	NA	-4.42	8.54E-10	-4.42	6.61E-10	0.00	1.00E+00
386	G__Sphingomonas	0.30	NA	0.00	1.00E+00	-4.73	1.14E-09	-4.73	6.69E-10	0.00	1.00E+00
737	G__Acidovorax	NA	NA	-1.04	4.81E-01	-5.09	4.12E-13	-4.05	8.57E-10	0.00	1.00E+00
525	G__Sphingomonas	1.71	NA	0.55	8.73E-01	-3.45	1.42E-07	-4.00	1.62E-09	1.09	9.52E-01
1002	G__Variovorax	NA	NA	0.00	NA	-4.63	3.67E-08	-4.63	2.20E-08	0.00	1.00E+00
888	G__Pseudorhodobacter	0.06	NA	0.00	1.00E+00	-4.19	9.19E-08	-4.19	6.32E-08	0.00	1.00E+00
1237	G__Pedobacter	NA	NA	0.00	NA	-3.83	1.30E-07	-3.83	1.18E-07	0.00	1.00E+00
482	F__Burkholderiaceae	0.61	NA	0.94	6.50E-01	-3.77	1.08E-04	-4.71	1.34E-07	1.81	5.04E-01
763	G__Rhizobacter	-0.84	NA	0.00	1.00E+00	-3.96	7.70E-07	-3.96	1.08E-06	0.00	1.00E+00
345	F__Burkholderiaceae	-0.39	7.55E-01	1.39	3.74E-01	-2.92	3.56E-03	-4.31	1.24E-06	2.73	8.82E-02
905	G__Lacihabitans	0.28	NA	0.51	1.00E+00	-3.37	7.25E-05	-3.88	1.60E-06	-1.00	1.00E+00
916	G__Hydrogenophaga	0.17	NA	0.00	1.00E+00	-3.61	1.13E-06	-3.62	1.61E-06	0.00	1.00E+00
1235	G__Allorhizobium	-0.06	NA	0.00	1.00E+00	-4.02	1.25E-06	-4.02	1.96E-06	0.00	1.00E+00
851	G__Arcicella	-0.58	NA	0.00	1.00E+00	-3.62	1.79E-06	-3.62	1.96E-06	0.00	1.00E+00
940	G__Brevundimonas	0.94	NA	1.37	3.59E-01	-2.60	7.91E-03	-3.97	2.24E-06	-2.67	7.08E-02
474	G__Sphingomonas	-1.49	NA	-0.29	1.00E+00	-3.60	2.60E-07	-3.31	2.46E-06	1.46	5.42E-01
1117	G__Roseomonas	0.00	NA	0.00	1.00E+00	-3.43	8.67E-06	-3.43	7.13E-06	0.00	1.00E+00
790	G__Arcicella	-0.97	NA	0.00	1.00E+00	-3.22	1.04E-05	-3.22	8.85E-06	0.00	1.00E+00
419	G__Sphingomonas	-1.20	NA	-0.14	1.00E+00	-3.45	6.88E-06	-3.31	1.30E-05	1.32	8.46E-01
288	F__Burkholderiaceae	-0.06	NA	0.00	1.00E+00	-3.84	5.73E-05	-3.84	1.38E-05	0.00	1.00E+00
742	G__Rhodoferax	0.64	NA	0.00	1.00E+00	-3.48	7.07E-05	-3.48	1.65E-05	0.00	1.00E+00
982	F__Sphingomonadaceae	0.00	NA	0.00	1.00E+00	-3.43	8.15E-05	-3.43	9.43E-05	0.00	1.00E+00
677	G__Sphingomonas	-1.97	NA	1.28	4.59E-01	-2.47	1.30E-02	-3.75	1.16E-04	2.49	1.15E-01
889	G__Allorhizobium	NA	NA	-0.79	7.73E-01	-4.48	2.41E-07	-3.68	1.55E-04	0.00	1.00E+00
720	F__Saccharimonadaceae	-1.49	NA	0.50	1.00E+00	-2.86	2.31E-03	-3.36	5.15E-04	1.00	1.00E+00
967	G__Acinetobacter	2.08	NA	0.80	6.63E-01	-2.58	7.13E-03	-3.38	5.33E-04	-1.59	5.22E-01
246	G__Arcicella	-1.72	9.56E-02	-0.66	8.23E-01	-4.06	1.13E-06	-3.40	5.89E-04	2.21	2.35E-01
767	G__Sphingomonas	-0.87	NA	1.23	4.62E-01	-2.10	4.17E-02	-3.33	1.90E-03	-2.43	1.15E-01
195	G__Pseudorhodobacter	-1.60	NA	-1.60	1.72E-01	-4.56	4.93E-08	-2.96	1.95E-03	0.00	1.00E+00
223	O__Rhizobiales	1.44	NA	0.00	1.00E+00	-2.96	1.25E-03	-2.96	2.45E-03	0.00	1.00E+00
559	G__Sphaerotilus	-1.04	2.63E-01	-1.67	1.60E-01	-4.55	2.76E-09	-2.88	2.52E-03	0.00	1.00E+00
966	F__Rhodobacteraceae	-1.66	NA	0.00	1.00E+00	-2.68	1.45E-03	-2.68	2.66E-03	0.00	1.00E+00
729	G__Emticicia	NA	NA	-1.80	1.06E-01	-4.54	2.13E-09	-2.73	2.73E-03	0.00	1.00E+00
632	O__Candidatus Moranbacteria	-1.32	NA	0.00	NA	-2.48	2.92E-03	-2.48	2.92E-03	0.00	1.00E+00
167	G__Sphingomonas	-0.92	3.29E-01	-0.28	1.00E+00	-3.02	9.31E-04	-2.74	5.11E-03	2.63	5.59E-02

833	F__Burkholderiaceae	-0.39	NA	-0.64	8.50E-01	-3.57	1.13E-04	-2.92	5.41E-03	1.39	9.24E-01
504	G__Acidovorax	1.87	5.27E-02	-0.85	6.58E-01	-3.74	1.92E-05	-2.89	5.78E-03	1.09	1.00E+00
512	G__Acinetobacter	-0.74	NA	0.00	1.00E+00	-2.49	4.21E-03	-2.49	8.97E-03	0.00	1.00E+00
727	F__Burkholderiaceae	0.31	NA	-0.34	1.00E+00	-3.32	1.30E-03	-2.97	1.39E-02	-2.29	2.46E-01
901	F__Beijerinckiaceae	NA	NA	0.00	NA	-2.76	5.34E-03	-2.76	1.39E-02	0.00	1.00E+00
1192	G__Aquabacterium	0.90	NA	-0.97	5.98E-01	-3.60	1.16E-04	-2.63	1.64E-02	0.00	1.00E+00
757	G__Hyphomicrobium	NA	NA	0.00	NA	-2.17	8.23E-03	-2.17	1.66E-02	0.00	1.00E+00
750	F__Burkholderiaceae	-1.87	NA	-2.28	5.34E-02	-4.77	3.64E-08	-2.49	1.68E-02	0.00	1.00E+00
269	F__Burkholderiaceae	-0.67	5.29E-01	-0.32	1.00E+00	-3.02	3.80E-03	-2.70	1.68E-02	2.89	5.34E-02
584	G__Acidovorax	-0.60	6.32E-01	0.00	1.00E+00	-2.04	1.99E-02	-2.04	3.45E-02	0.00	1.00E+00
712	G__Acidovorax	-1.58	1.27E-01	0.00	1.00E+00	-2.15	2.35E-02	-2.15	4.01E-02	0.00	1.00E+00
291	G__Brevundimonas	0.17	9.86E-01	-0.23	1.00E+00	-2.69	1.10E-02	-2.45	4.07E-02	-1.92	3.26E-01
84	G__Deefgea	-0.06	NA	-5.38	4.84E-11	-3.19	2.93E-03	2.19	4.51E-02	0.00	1.00E+00
294	G__Sphaerotilus	-2.60	2.58E-04	-2.88	4.14E-03	-4.38	7.70E-07	-1.50	3.37E-01	0.00	1.00E+00
177	G__Deefgea	NA	NA	-3.66	8.79E-05	-2.36	3.97E-02	1.30	4.61E-01	0.00	1.00E+00
318	G__Flavobacterium	-1.19	2.30E-01	-3.26	8.72E-04	-2.69	8.23E-03	0.57	9.99E-01	0.00	1.00E+00
486	F__Burkholderiaceae	-3.39	NA	-2.45	3.72E-02	-2.45	3.45E-02	0.00	NA	-1.59	7.00E-01
841	G__Limnohabitans	-0.34	7.92E-01	0.00	1.00E+00	-2.00	2.49E-02	-2.00	4.54E-02	0.00	1.00E+00
520	F__Rhizobiales Incertae Sedis	1.97	NA	0.00	NA	-2.01	3.09E-02	-2.01	5.51E-02	0.00	1.00E+00
123	F__Holosporaceae	0.41	NA	0.00	NA	-1.99	3.29E-02	-1.99	5.86E-02	0.00	1.00E+00
424	G__Pseudomonas	NA	NA	-2.00	1.58E-01	-4.38	1.39E-05	-2.39	1.00E-01	0.00	1.00E+00
1208	G__Allorhizobium	NA	NA	-1.37	3.65E-01	-3.40	6.82E-04	-2.03	1.06E-01	0.00	1.00E+00
1135	G__Brevundimonas	0.06	NA	-0.42	1.00E+00	-2.68	1.76E-02	-2.26	1.06E-01	2.52	1.78E-01
740	G__Flectobacillus	-0.70	NA	-1.52	2.36E-01	-3.08	3.52E-03	-1.55	3.16E-01	-0.91	1.00E+00
692	G__Pantoea	NA	NA	-1.90	1.09E-01	-3.10	3.80E-03	-1.20	5.20E-01	0.00	1.00E+00
799	G__Lawsonella	2.64	6.52E-05	-1.49	2.13E-01	-2.60	9.91E-03	-1.10	5.25E-01	0.00	1.00E+00
925	G__Allorhizobium	0.30	NA	-1.10	4.85E-01	-2.31	4.58E-02	-1.21	5.38E-01	-1.81	4.65E-01
1239	F__Burkholderiaceae	0.00	NA	-1.36	2.94E-01	-2.43	2.28E-02	-1.07	6.18E-01	0.00	1.00E+00
913	G__Acinetobacter	-0.65	NA	-1.32	3.49E-01	-2.17	3.97E-02	-0.84	8.28E-01	0.00	1.00E+00
77	G__Aeromonas	0.84	NA	-2.10	5.45E-02	2.67	1.88E-02	4.77	3.70E-08	1.50	5.53E-01
87	G__Burkholderia	1.95	NA	-2.48	5.20E-02	4.51	1.04E-05	7.00	1.59E-14	0.63	1.00E+00
103	G__Buttiauxella	NA	NA	-0.03	NA	4.93	3.62E-08	4.96	1.65E-09	-0.91	1.00E+00
112	G__Pseudomonas	NA	NA	0.92	5.03E-01	4.28	8.05E-10	3.36	9.48E-06	0.84	1.00E+00
119	F__Enterobacteriaceae	NA	NA	0.57	8.54E-01	4.30	4.97E-08	3.73	7.19E-06	-0.09	1.00E+00
142	G__Psychrobacter	-0.70	NA	0.03	NA	3.96	3.99E-04	3.93	5.89E-04	2.09	2.49E-01
150	F__Enterobacteriaceae	NA	NA	0.47	8.39E-01	6.79	1.98E-23	6.32	3.09E-20	-0.12	1.00E+00
151	G__Buttiauxella	NA	NA	0.23	NA	5.29	1.77E-09	5.05	3.09E-08	-0.82	1.00E+00
152	F__Paracaedibacteraceae	3.66	3.18E-07	-0.98	4.12E-01	5.72	4.80E-16	6.69	6.56E-22	0.24	1.00E+00
168	F__Enterobacteriaceae	NA	NA	1.31	3.38E-01	6.42	2.52E-14	5.12	1.06E-08	0.67	1.00E+00
176	G__Burkholderia	1.06	NA	-2.56	3.38E-02	4.13	7.11E-04	6.69	7.84E-13	0.75	1.00E+00
179	G__Legionella	-6.78	2.06E-39	-1.99	5.82E-02	2.52	2.86E-02	4.51	5.87E-07	-0.06	1.00E+00
215	F__Betaproteobacteriales Incertae	1.69	NA	1.12	5.48E-01	4.83	5.61E-06	3.71	5.71E-03	0.51	1.00E+00
240	O__Betaproteobacteriales	-0.46	NA	-2.10	5.96E-02	3.78	9.13E-04	5.88	1.54E-11	0.00	1.00E+00

327	F__Enterobacteriaceae	NA	NA	1.65	1.26E-01	5.14	1.55E-10	3.49	9.91E-04	0.01	1.00E+00
349	F__Enterobacteriaceae	NA	NA	0.09	NA	5.33	6.96E-10	5.24	5.37E-10	0.53	1.00E+00
358	G__Mycobacterium	NA	NA	-0.45	1.00E+00	3.55	1.28E-03	4.00	1.63E-04	0.61	1.00E+00
404	F__Betaproteobacteriales Incertae	0.00	NA	1.85	1.58E-01	4.92	1.10E-06	3.07	1.98E-02	0.18	1.00E+00
442	G__Citrobacter	0.06	NA	0.14	NA	4.05	3.38E-04	3.91	8.86E-04	1.25	9.47E-01
480	G__Serratia	NA	NA	-1.03	5.26E-01	4.26	1.00E-04	5.29	2.88E-08	0.73	1.00E+00
542	F__Betaproteobacteriales Incertae	0.00	NA	-0.05	NA	4.51	8.86E-06	4.56	7.03E-06	0.29	1.00E+00
581	F__Enterobacteriaceae	NA	NA	0.85	5.95E-01	4.24	1.94E-06	3.40	2.51E-03	-1.73	3.27E-01
612	F__Enterobacteriaceae	NA	NA	1.64	1.18E-01	5.15	7.66E-10	3.51	2.10E-03	0.26	1.00E+00
693	G__Rudanella	NA	NA	1.47	2.33E-01	4.16	5.73E-05	2.69	4.51E-02	-1.06	9.90E-01
702	G__Fusobacterium	-0.35	NA	0.80	6.59E-01	3.90	6.36E-04	3.10	1.54E-02	-1.68	4.49E-01
704	G__Nevskia	NA	NA	0.56	8.92E-01	4.26	6.41E-06	3.70	9.91E-04	-0.11	1.00E+00
845	G__Flavobacterium	NA	NA	0.10	NA	3.88	6.04E-04	3.79	9.60E-04	-0.28	1.00E+00
13	F__Mycoplasmataceae	0.63	3.70E-01	2.92	1.12E-03	2.35	1.20E-02	-0.56	9.99E-01	-0.85	1.00E+00
15	G__Plesiomonas	1.08	1.62E-01	5.09	5.36E-08	3.14	4.21E-03	-1.95	2.90E-01	-1.61	5.41E-01
16	F__Mycoplasmataceae	0.64	3.41E-01	3.23	2.77E-04	2.12	2.45E-02	-1.11	6.90E-01	-0.48	1.00E+00
17	G__Plesiomonas	0.89	1.83E-01	4.34	5.86E-06	3.40	1.30E-03	-0.93	8.97E-01	-1.50	6.50E-01
82	F__Clostridiaceae 1	1.55	NA	3.39	3.24E-04	2.15	3.85E-02	-1.24	5.78E-01	-0.19	1.00E+00
95	F__Neisseriaceae	NA	NA	8.99	2.26E-34	9.00	2.14E-34	0.00	NA	0.46	1.00E+00
162	G__Pseudomonas	-0.11	NA	3.75	8.62E-06	2.96	1.32E-03	-0.79	9.63E-01	-0.53	1.00E+00
234	G__Psychrobacter	NA	NA	4.10	1.15E-06	4.10	1.47E-07	0.00	NA	0.28	1.00E+00
266	G__Staphylococcus	0.25	NA	3.04	7.96E-03	2.67	2.82E-02	-0.37	NA	-1.22	9.52E-01
293	G__MD3-55	0.23	NA	5.15	6.19E-16	5.70	5.01E-18	0.55	NA	0.52	1.00E+00
301	G__Pseudomonas	-2.03	9.57E-03	3.33	5.52E-04	5.00	1.96E-09	1.68	3.78E-01	0.44	1.00E+00
362	G__Acinetobacter	0.06	NA	2.59	3.15E-03	2.58	6.90E-03	0.00	NA	-1.79	2.00E-01
373	G__Hydrogenophaga	NA	NA	2.95	8.34E-03	4.58	1.98E-06	1.63	4.58E-01	-1.80	4.55E-01
575	G__Streptococcus	-1.12	2.64E-01	2.37	3.84E-03	2.27	9.27E-03	-0.10	NA	0.22	1.00E+00
766	G__Rhizobacter	NA	NA	2.80	3.36E-03	4.49	2.64E-07	1.68	3.85E-01	-0.77	1.00E+00
855	F__Beijerinckiaceae	NA	NA	2.10	2.46E-02	2.10	2.36E-02	0.00	NA	-1.03	7.41E-01
661	G__Sphingomonas	-0.76	NA	2.20	5.22E-02	2.51	2.29E-02	0.31	NA	-1.19	9.38E-01
601	G__Acinetobacter	1.31	NA	2.10	6.51E-02	3.01	6.76E-03	0.91	8.92E-01	0.14	1.00E+00
893	F__Kineosporiaceae	NA	NA	1.84	6.59E-02	4.16	1.81E-06	2.33	8.26E-02	-0.01	1.00E+00
217	G__Acinetobacter	0.07	NA	2.25	6.59E-02	2.94	1.76E-02	0.68	9.99E-01	-0.09	1.00E+00
309	G__Acinetobacter	0.63	5.92E-01	1.72	1.20E-01	3.03	4.12E-03	1.30	5.45E-01	0.54	1.00E+00
1155	G__Duganella	-0.84	NA	1.41	2.09E-01	3.27	1.06E-03	1.86	2.46E-01	1.26	6.32E-01
918	G__Flavobacterium	NA	NA	1.38	2.16E-01	3.56	6.16E-04	2.18	1.46E-01	0.69	1.00E+00
2	F__Mycoplasmataceae	0.44	4.47E-01	1.43	2.75E-01	3.18	3.55E-03	1.75	2.76E-01	-0.70	1.00E+00
686	G__Prevotella	-0.03	NA	1.21	3.23E-01	2.22	3.60E-02	1.01	8.07E-01	-1.43	5.68E-01
43	F__Clostridiaceae 1	0.39	7.63E-01	1.30	4.01E-01	3.39	3.00E-03	2.09	2.03E-01	-0.91	1.00E+00
212	G__Staphylococcus	-1.04	3.44E-01	1.24	4.13E-01	2.68	1.85E-02	1.44	4.87E-01	2.13	2.14E-01
157	F__Enterobacteriaceae	-0.26	8.82E-01	1.19	5.03E-01	3.77	1.18E-03	2.58	8.36E-02	-1.88	4.98E-01
89	F__Clostridiaceae 1	-0.89	2.67E-01	1.04	5.06E-01	2.40	3.23E-02	1.36	4.94E-01	1.02	1.00E+00
92	F__Clostridiaceae 1	-0.67	5.46E-01	0.85	5.62E-01	2.85	4.12E-03	1.99	1.50E-01	-0.38	1.00E+00

391	F__Enterobacteriaceae	1.54	NA	0.85	5.66E-01	2.90	5.69E-03	2.05	1.53E-01	1.89	2.33E-01
452	G__Flavobacterium	-3.15	NA	0.88	6.72E-01	2.85	2.43E-02	1.98	2.67E-01	0.52	1.00E+00
248	G__Rudanella	-0.87	NA	0.79	7.98E-01	2.64	4.65E-02	1.85	3.59E-01	-0.70	1.00E+00
844	G__Sphingobacterium	-0.70	NA	0.61	8.83E-01	2.79	2.23E-02	2.18	1.65E-01	0.20	1.00E+00
10	F__Mycoplasmataceae	0.34	6.35E-01	0.38	1.00E+00	2.58	1.28E-02	2.20	8.18E-02	-0.91	1.00E+00
638	G__Kocuria	-2.46	NA	0.06	NA	2.28	2.86E-02	2.22	7.52E-02	0.95	1.00E+00
371	G__Streptococcus	-0.92	3.93E-01	4.27	3.31E-05	0.10	NA	-4.17	4.83E-04	-0.32	1.00E+00
160	G__Lawsonella	2.34	3.09E-05	3.78	2.77E-04	0.34	1.00E+00	-3.44	2.73E-03	0.74	1.00E+00
662	G__Gordonia	-2.43	NA	3.15	3.09E-03	-0.15	NA	-3.30	5.77E-03	1.97	2.63E-01
300	G__Rhodococcus	-1.20	NA	2.76	1.21E-02	-1.21	4.08E-01	-3.97	1.26E-04	-0.04	1.00E+00
222	G__Acinetobacter	-0.42	7.56E-01	2.80	2.02E-02	-1.34	3.07E-01	-4.14	8.61E-05	-1.78	4.16E-01
185	G__Rhodococcus	2.45	NA	2.29	6.03E-02	-0.97	7.38E-01	-3.25	6.03E-03	0.17	1.00E+00
754	G__Rhodoferax	0.00	NA	2.26	6.51E-02	-1.32	3.90E-01	-3.57	2.49E-03	0.54	1.00E+00
241	G__Rhodoferax	1.65	8.60E-02	2.26	6.59E-02	-1.17	5.46E-01	-3.43	4.82E-03	-0.13	1.00E+00
237	G__Rhodococcus	1.02	NA	2.11	8.18E-02	-1.92	9.43E-02	-4.03	9.43E-05	-0.21	1.00E+00
85	G__Rhodococcus	-0.89	3.62E-01	1.61	8.74E-02	-0.64	8.37E-01	-2.24	2.00E-02	0.74	1.00E+00
1057	O__Saccharimonadales	1.15	NA	1.81	1.26E-01	-1.50	1.93E-01	-3.30	2.55E-03	0.21	1.00E+00
267	G__Rhodococcus	-0.09	NA	1.75	1.49E-01	-0.73	8.38E-01	-2.48	4.48E-02	0.76	1.00E+00
1	F__Mycoplasmataceae	0.62	5.64E-01	1.77	2.33E-01	-2.54	5.22E-02	-4.31	4.32E-04	-0.05	1.00E+00
724	G__Brevundimonas	-1.37	1.52E-01	1.32	3.11E-01	-0.95	5.44E-01	-2.28	3.96E-02	-2.59	5.34E-02
503	G__Corynebacterium 1	1.75	NA	1.61	3.74E-01	-1.69	2.52E-01	-3.30	1.74E-02	0.84	1.00E+00
1212	G__Herbaspirillum	NA	NA	1.10	4.77E-01	-1.53	1.31E-01	-2.63	1.48E-02	-2.17	1.83E-01
669	G__Pectobacterium	-0.84	NA	0.90	5.90E-01	-1.88	5.55E-02	-2.78	5.78E-03	1.75	3.55E-01
708	G__Cutibacterium	-0.26	NA	0.61	8.08E-01	-1.73	6.16E-02	-2.33	1.95E-02	-1.64	3.98E-01
36	G__Aeromonas	1.41	NA	-5.01	9.99E-12	-0.47	9.66E-01	4.54	1.92E-10	-2.43	1.15E-01
79	G__Aeromonas	1.23	NA	-4.80	2.46E-10	-0.98	6.88E-01	3.82	1.42E-07	0.00	1.00E+00
52	G__Aeromonas	NA	NA	-4.58	4.84E-11	0.00	NA	4.59	1.89E-11	0.00	1.00E+00
149	G__Aeromonas	NA	NA	-4.52	1.41E-08	-0.95	7.38E-01	3.57	1.38E-05	1.09	1.00E+00
634	G__Chryseobacterium	NA	NA	-4.29	1.62E-06	-1.92	1.01E-01	2.37	2.92E-02	0.00	1.00E+00
76	G__Aeromonas	NA	NA	-4.15	7.27E-08	0.00	NA	4.15	3.75E-08	0.00	1.00E+00
458	G__Undibacterium	-1.09	NA	-5.49	3.10E-17	-0.86	7.26E-01	4.63	1.18E-14	0.00	1.00E+00
489	G__Flavobacterium	0.80	NA	-4.03	2.27E-07	-1.12	4.35E-01	2.91	1.03E-03	0.00	1.00E+00
238	G__Aeromonas	NA	NA	-3.36	1.22E-05	0.00	NA	3.36	1.27E-05	0.00	1.00E+00
623	C__Gracilibacteria	NA	NA	-3.20	1.40E-04	0.00	NA	3.19	5.23E-04	0.00	1.00E+00
226	G__Aeromonas	0.00	NA	-3.10	1.16E-03	-0.23	NA	2.86	1.03E-02	1.70	5.22E-01
585	G__Candidatus Megaira	NA	NA	-2.77	3.15E-03	-0.20	NA	2.56	1.74E-02	-2.17	2.19E-01
71	G__Deefgea	NA	NA	-2.39	9.08E-03	1.38	3.09E-01	3.76	9.30E-06	2.67	7.11E-02
655	G__Stenotrophomonas	NA	NA	-2.22	1.18E-02	0.00	NA	2.22	3.26E-02	0.00	1.00E+00
245	G__Pseudomonas	-1.50	NA	-2.05	5.60E-02	0.87	7.38E-01	2.92	5.03E-03	0.70	1.00E+00
481	G__Pseudomonas	1.15	NA	-1.98	6.98E-02	1.20	5.00E-01	3.18	5.77E-03	0.89	1.00E+00
635	G__Pseudomonas	0.36	NA	-1.75	1.58E-01	2.00	1.21E-01	3.76	1.07E-03	-0.38	1.00E+00
209	G__Deefgea	NA	NA	-1.65	8.57E-02	0.75	8.26E-01	2.40	1.98E-02	1.84	2.74E-01
549	G__Chryseobacterium	-2.75	NA	-1.58	8.88E-02	1.07	5.29E-01	2.65	1.05E-02	2.09	1.87E-01

83	G__Verticia	-1.34	2.02E-01	-1.37	3.13E-01	1.28	3.56E-01	2.65	1.97E-02	-1.26	9.38E-01
45	G__Verticia	-1.24	2.32E-01	-1.29	3.74E-01	1.41	2.41E-01	2.70	1.67E-02	-1.37	8.00E-01
1020	G__Lysobacter	-0.80	NA	-1.24	3.05E-01	1.13	4.46E-01	2.38	3.95E-02	2.21	1.61E-01
996	F__Burkholderiaceae	NA	NA	-1.09	4.70E-01	1.74	1.57E-01	2.83	1.52E-02	1.83	3.18E-01
50	G__Staphylococcus	-1.67	NA	-0.52	1.00E+00	2.55	5.51E-02	3.07	2.95E-02	0.62	1.00E+00
333	G__Anaerococcus	-0.26	9.29E-01	3.59	2.32E-04	1.98	5.78E-02	-1.61	4.02E-01	-0.52	1.00E+00
260	G__Cutibacterium	0.46	6.63E-01	2.92	1.52E-03	1.06	4.24E-01	-1.86	1.62E-01	-0.28	1.00E+00
8	G__Plesiomonas	0.99	1.67E-01	3.36	2.91E-03	0.67	8.86E-01	-2.69	6.20E-02	-3.02	6.89E-02
698	G__Streptococcus	-3.36	NA	3.14	3.02E-03	1.05	5.74E-01	-2.09	2.00E-01	0.59	1.00E+00
175	G__Vibrionimonas	0.11	NA	2.71	6.71E-03	0.87	7.54E-01	-1.84	2.20E-01	-1.02	1.00E+00
352	G__Herbaspirillum	1.03	2.98E-01	3.04	7.96E-03	0.71	8.86E-01	-2.33	1.50E-01	-0.72	1.00E+00
265	G__Staphylococcus	1.45	1.54E-01	2.87	8.84E-03	0.26	1.00E+00	-2.61	5.11E-02	1.58	5.89E-01
428	G__Stenotrophomonas	-0.88	4.36E-01	2.50	2.53E-02	1.87	1.04E-01	-0.63	9.99E-01	1.71	4.36E-01
110	G__Staphylococcus	0.60	5.02E-01	2.30	4.87E-02	0.35	1.00E+00	-1.95	2.06E-01	-1.31	9.24E-01
320	G__Aeromonas	NA	NA	-2.55	3.92E-03	-1.36	2.67E-01	1.20	4.61E-01	0.00	1.00E+00
713	G__Acinetobacter	-1.77	NA	-2.82	3.93E-03	-1.84	1.31E-01	0.98	8.04E-01	0.00	1.00E+00
189	G__Deefgea	NA	NA	-2.40	1.30E-02	-0.97	6.30E-01	1.43	3.24E-01	0.00	1.00E+00
399	G__Nocardioides	-4.09	4.01E-09	0.63	8.54E-01	2.25	5.93E-02	1.62	4.02E-01	-0.30	1.00E+00
735	G__Sphaerotilus	-4.07	4.01E-09	NA	NA	NA	NA	NA	NA	NA	NA
532	G__Candidatus Trichorickettsia	-3.97	1.16E-09	0.10	NA	-0.09	NA	-0.19	NA	0.19	NA
376	G__Sphingomonas	-3.96	4.73E-08	-1.32	3.74E-01	-1.31	3.50E-01	0.02	NA	-0.42	1.00E+00
421	F__Burkholderiaceae	-3.75	6.88E-08	NA	NA	NA	NA	NA	NA	NA	NA
764	G__Janthinobacterium	-3.75	3.84E-08	0.10	NA	0.00	NA	-0.09	NA	0.00	1.00E+00
629	F__Amoebophilaceae	-3.68	1.15E-07	NA	NA	NA	NA	NA	NA	NA	NA
981	G__Janthinobacterium	-3.49	2.76E-07	-0.10	NA	-0.10	NA	0.00	NA	-0.19	NA
171	G__Janthinobacterium	-2.84	4.04E-05	-0.55	9.12E-01	1.28	3.06E-01	1.83	1.88E-01	0.62	1.00E+00
422	G__Alkanindiges	-2.83	2.00E-05	NA	NA	NA	NA	NA	NA	NA	NA
700	G__Sphaerotilus	-2.75	2.01E-05	-2.04	4.87E-02	-1.81	8.60E-02	0.23	NA	0.00	1.00E+00
962	G__Xylophilus	-2.25	2.20E-03	0.00	NA	-0.83	NA	-0.84	NA	0.00	NA
1039	G__Acidovorax	-2.17	3.35E-03	0.00	NA	-0.83	NA	-0.84	NA	0.00	NA
247	G__Acinetobacter	-2.16	5.56E-03	1.41	3.90E-01	-0.27	1.00E+00	-1.67	4.06E-01	0.06	1.00E+00
400	G__Rhodococcus	-1.93	1.24E-02	0.76	7.49E-01	-1.69	1.48E-01	-2.44	5.38E-02	2.00	2.64E-01
146	G__Cutibacterium	1.32	1.06E-02	-0.10	NA	-0.09	NA	0.00	NA	0.19	NA
74	G__Lawsonella	1.53	2.78E-08	1.59	2.17E-01	0.31	1.00E+00	-1.29	5.81E-01	0.37	1.00E+00
619	G__Lawsonella	2.19	1.15E-03	0.36	1.00E+00	-1.02	3.66E-01	-1.38	2.78E-01	-0.70	1.00E+00
914	G__Cutibacterium	2.38	2.24E-05	NA	NA	NA	NA	NA	NA	NA	NA
199	G__Arenimonas	2.39	1.14E-03	0.00	NA	0.11	NA	0.11	NA	0.00	NA
412	G__Exiguobacterium	2.39	2.20E-03	0.19	NA	0.00	NA	-0.19	NA	0.00	NA
1003	G__Exiguobacterium	3.50	8.05E-08	NA	NA	NA	NA	NA	NA	NA	NA
866	G__Chryseobacterium	4.51	2.02E-12	NA	NA	NA	NA	NA	NA	NA	NA

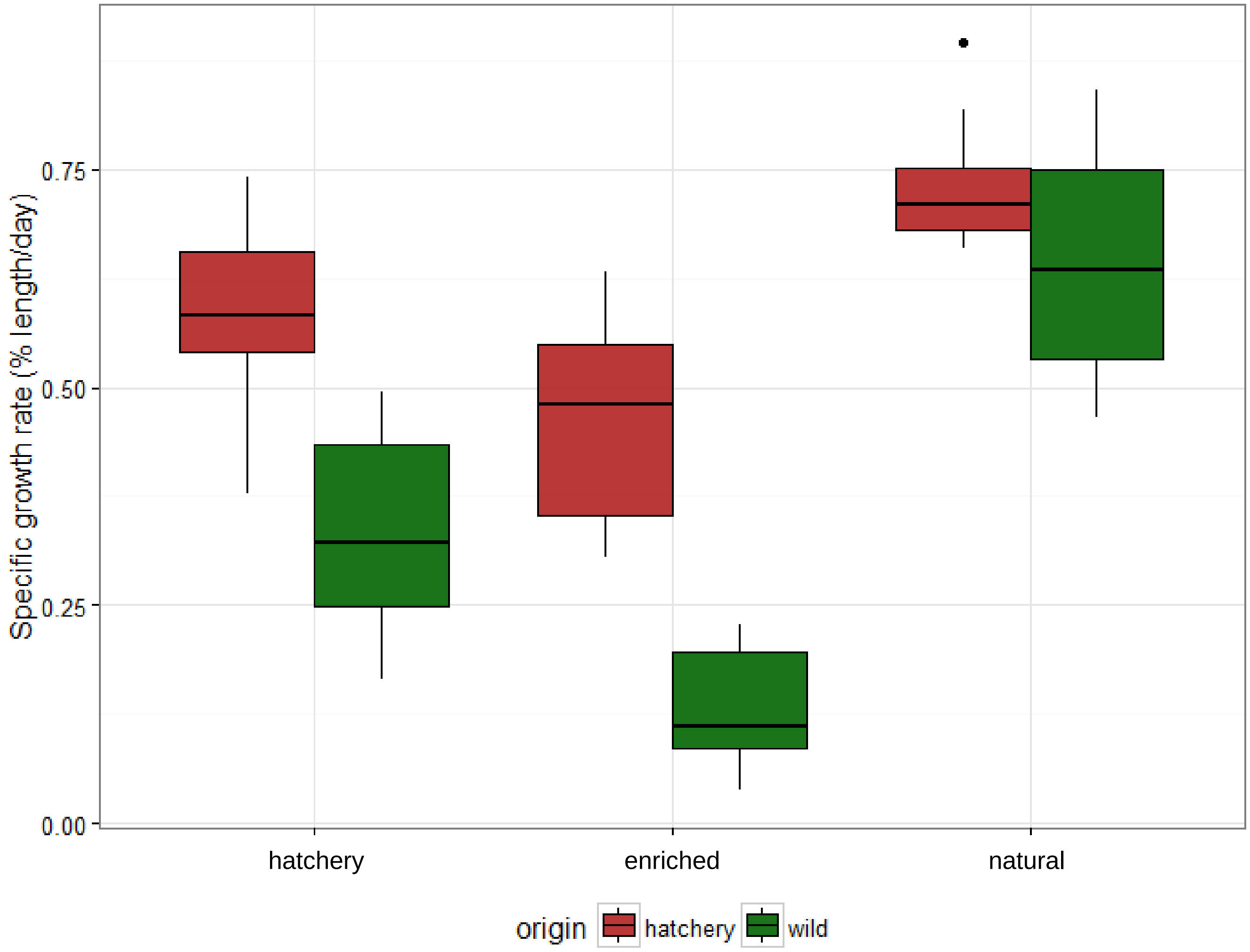

Figure S1. Specific growth rate for individually matched fish during the course of the experiment of fish in each treatment group (n=8).

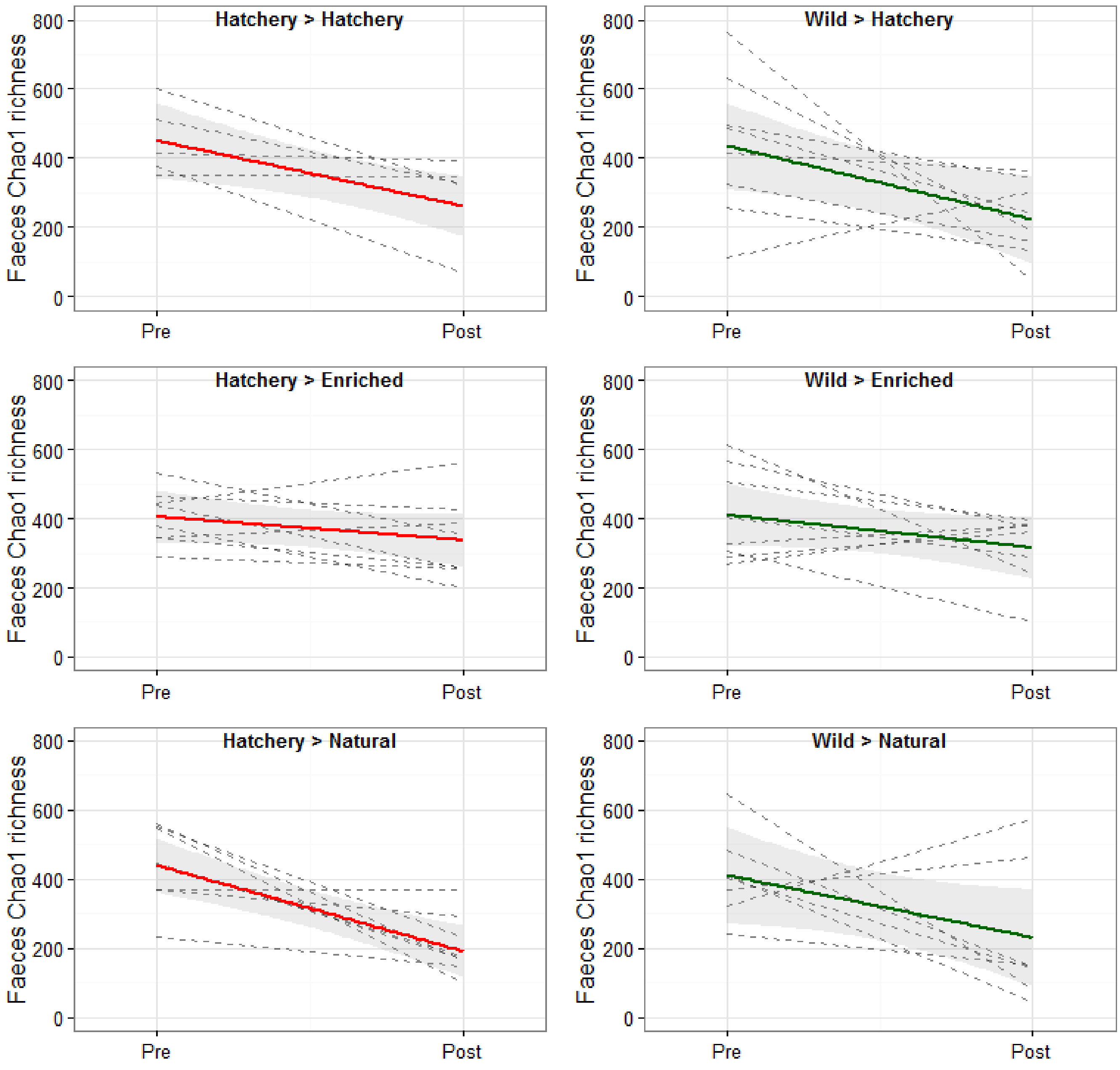

Figure S2. Change in faecal Chao1 richness for all matched fish during the course of the experiment, each dashed line represents an individual fish, with coloured lines displaying the average for each group (n=8) and grey shading indicating 95% confidence intervals.

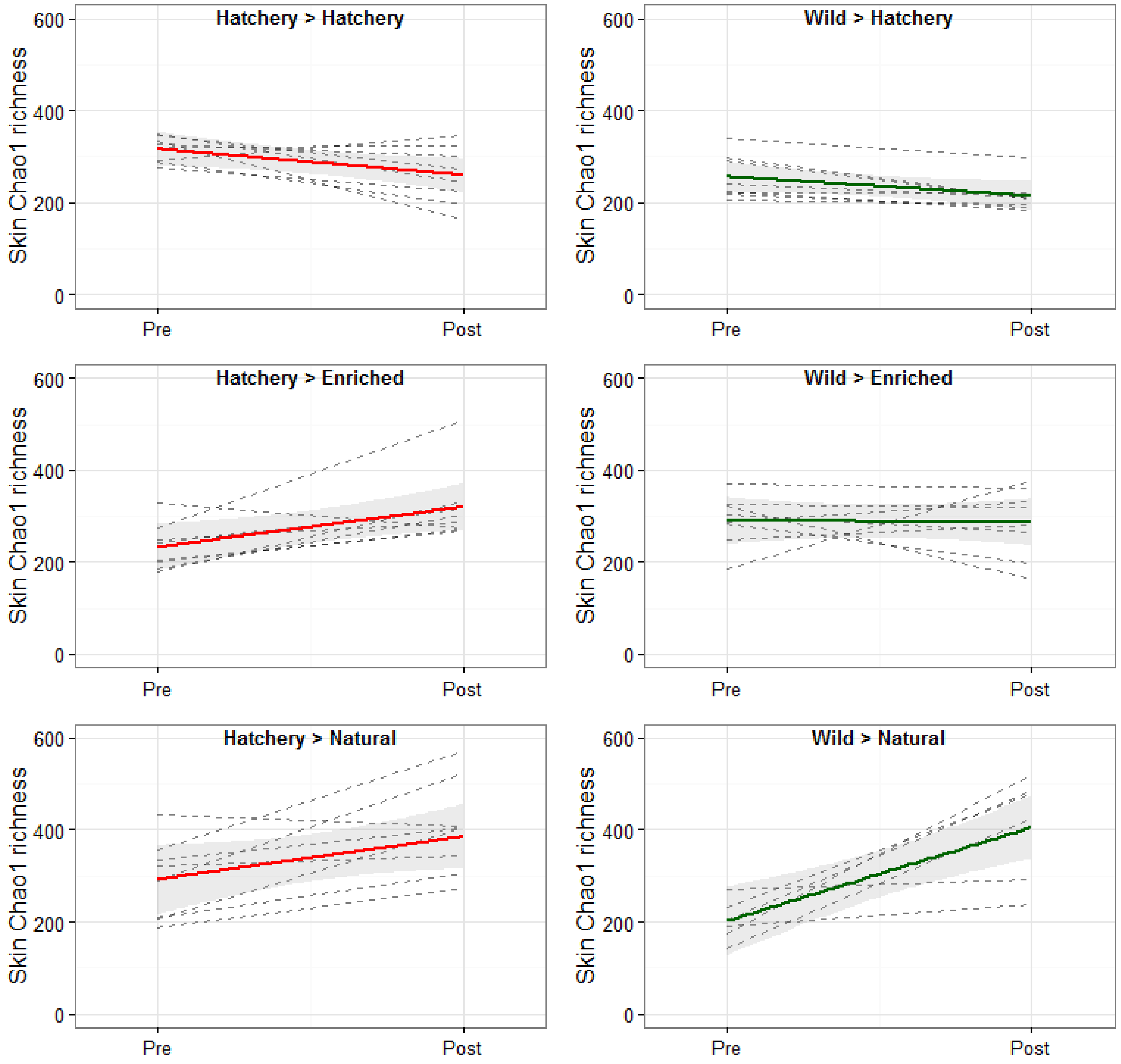

Figure S3. Change in skin Chao1 richness for all matched fish during the course of the experiment, each dashed line represents an individual fish, with coloured lines displaying the average for each group (n=8) and grey shading indicating 95% confidence intervals.

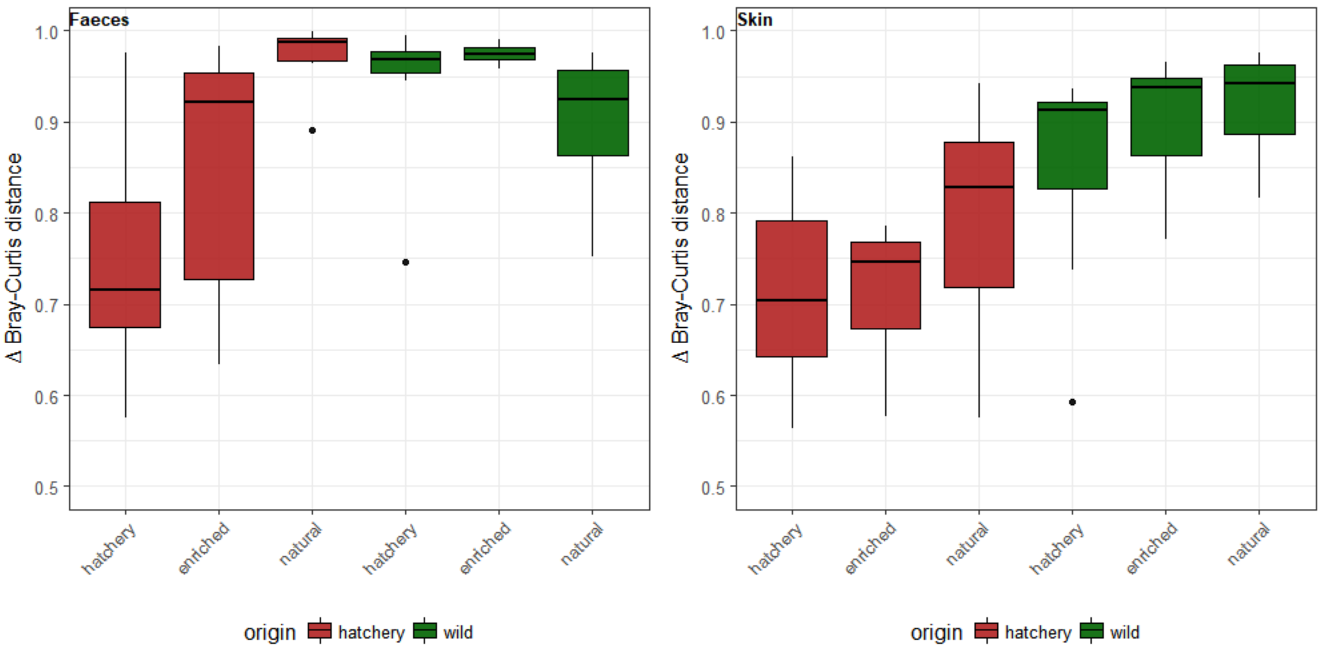

Figure S4. Change in microbiome structure for individually matched fish over the course of the experiment, based on Bray-Curtis distances, in each treatment group (n=8).

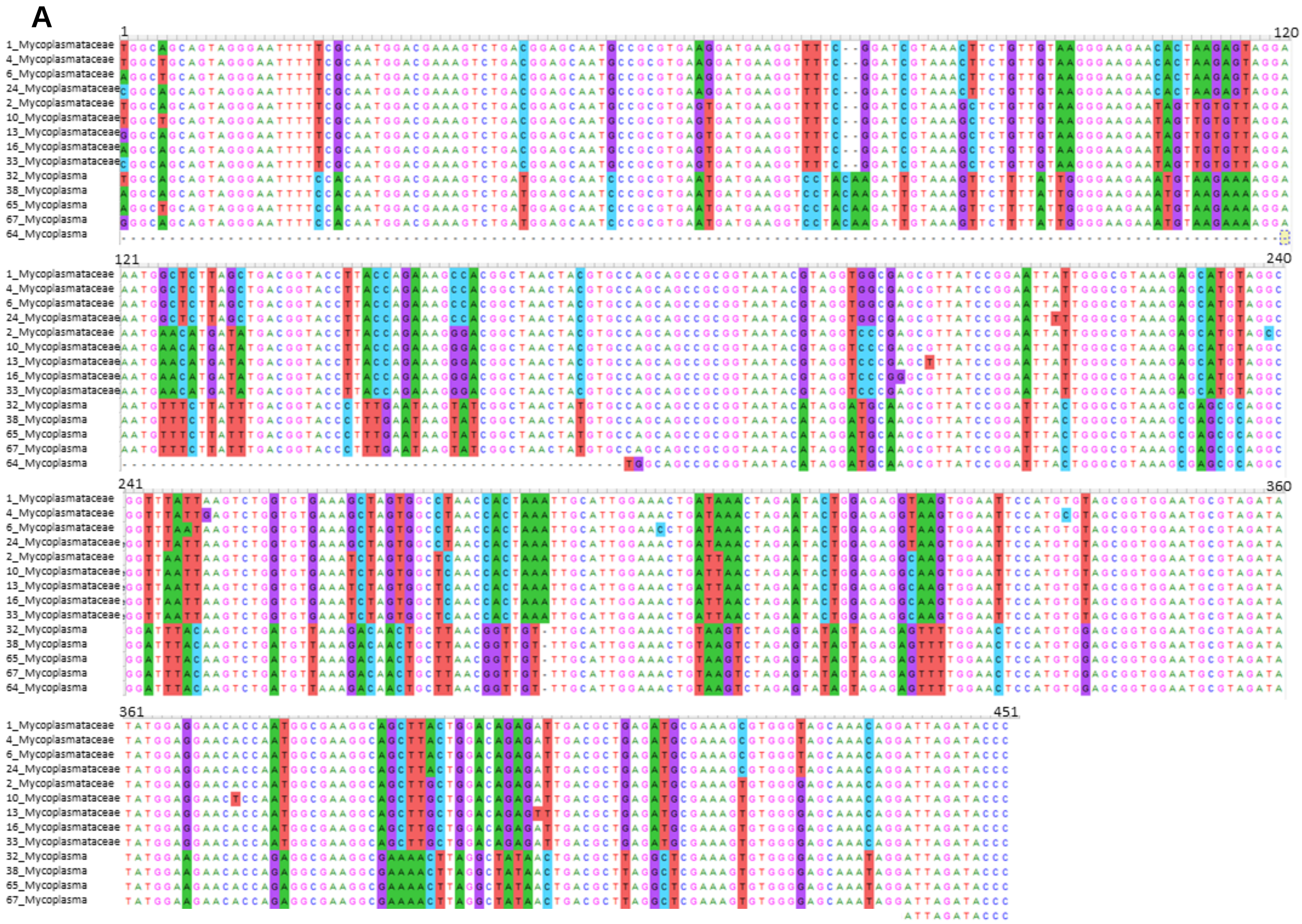

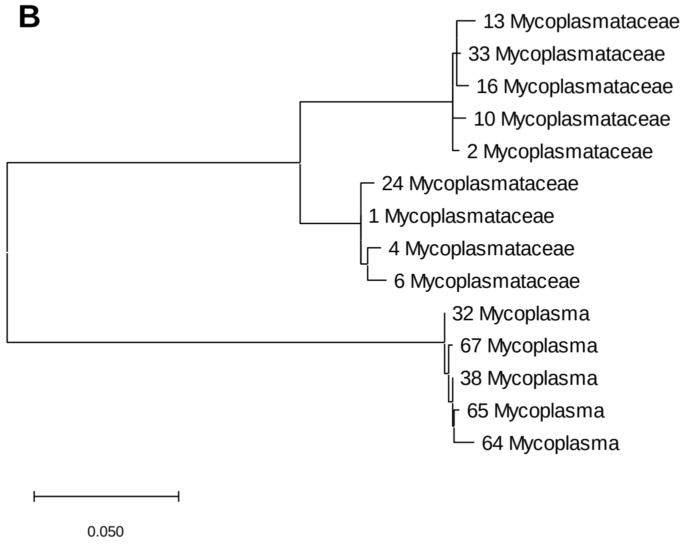

Figure S5. A) Sequence alignment and B) phylogenetic tree of faecal ASVs within the family Mycoplasmataceae. ASVs displayed are those within the 50 most abundant in the gut microbiome. ASVs 1,4,6 & 24 were prevalent in fish in the hatchery environment, while ASVs 2,10,13,16 & 33 were prevalent in the natural environment, both before and after translocation.
